# In search of better models for explaining atmospheric methane accumulation

**DOI:** 10.1093/af/vfaf001

**Published:** 2025-04-05

**Authors:** Peer Ederer, Taras Iliushyk

**Affiliations:** Global Observatory for Accurate Livestock Sciences, Switzerland; Global Observatory for Accurate Livestock Sciences, Switzerland

**Keywords:** atmospheric methane concentrations, empirical observations, methane models

**Implications**

- Methane is the third most important greenhouse gas after water vapor and carbon dioxide. However, the dynamics of methane in climate warming are even more contested than the ongoing peri-scientific controversy of GWP100 vs GWP* suggests, and many questions about methane dynamics remain unanswered (see also supplementary documentation to this article).
- Several core empirical observations are yet to be explained satisfactorily and unambiguously. For instance, why did methane concentrations begin to abate from the mid-1980s onwards and then stop rising between 2000 and 2006? Why did they thereafter resume the fastest rate of increase ever, even though overall emissions likely remained stable? Why has the fractionation ratio of C13/12 reversed its 100-year steep upward trend after this 2000–2006 period and is now falling rapidly? What is the reason for a robust 7-year cycle in atmospheric methane concentrations?
- Paleolithical empirical observations are also not well explained: How can the steep fall and rise of global methane concentrations during the Young Dryas (12,800 years ago), be explained? Why does the Northern Hemisphere have 8% higher methane concentrations than the South since the Young Dryas, until today? Did the Anthropocene already begin 5,000 years ago when methane levels started to rise against their typical pattern of the last 800,000 years? How can the explosive growth of atmospheric concentrations since the 1900s be explained, to begin with?
- While there are so many fundamental questions, methodological challenges, and imprecise modeling outputs, peri-scientific and occasionally even scientific discourse calls with certainty for methane emission reductions that appear premature in light of these uncertainties.
- The search for better models to explain the methane dynamics is not only a question of higher data resolution in observations. Empirical data density is already outpacing the capacity of the models. Paradigmatic, conceptual, and mathematical improvement of the models appears necessary as well, to do justice to the undisputed non-linear dynamics in the terrestrial-atmospheric methane cycle.
- The Supplementary Documentation to this article provides further details on where is the atmospheric methane, how much is emitted where and by what, where is it destroyed, what are isotope fractionation ratios, and which satellites are observing methane. It also provides an overview of some of the controversies around describing and calculating the impact of methane concentrations on climate change, starting with some definitions.

## Introduction

Methane is the third most important greenhouse gas after water vapor and carbon dioxide. Throughout the last 150 years, sciences could observe an ever-strengthening increase of methane in the atmosphere. The average atmospheric volume mixing ratio of surface methane (CH_4_) in 2023 was 1922 parts per billion of air molecules (ppb), which is 17% higher than 40 years previously in 1983 when detailed observations began, and close to three times the pre-industrial level of 729 ppb in the year 1750 (IPCC AR 6 Annex III, Table AIII. 1a, p 2141). On the assumption that atmospheric CH_4_ concentrations can be reduced, or at least be prevented from rising further, the Global Methane Pledge (GMP) was initiated at COP26 in 2021, aiming to reduce global methane emissions at least by 30% from 2020 levels by 2030 through voluntary actions. To date, 158 countries joined the GMP, representing a little more than 50% of all global emissions, with the notable exceptions of India, China, and Russia (https://www.globalmethanepledge.org/). This means that signing countries need to reduce their emissions by 60% to achieve this target. Ruminant livestock amount to 20% of the global emissions budget. In several signatory countries such as New Zealand, Australia, Ethiopia, Kenya, Uruguay, Argentina, Brazil, Mongolia, Ireland, and Switzerland, ruminants account for almost all of the respective national methane emissions. Fairly or unfairly, the ruminant livestock sector is therefore under immense scrutiny for how it can reduce its methane emissions (Tedeschi and Beauchemin, 2023).

In light of the threat of climate change, the rising atmospheric methane concentrations have triggered considerable scientific effort in increasing the observation points of empirical data collection and improving the atmospheric modeling tools with the purpose of understanding the causes of the fast rise of atmospheric methane, and consequently, define strategies that may lead to a decrease of atmospheric methane and thus abate climate change. Perhaps surprisingly, these efforts are relatively recent with high-resolution satellite data and powerful models available only for a few years (Supplementary Figure S5).

Thus paradigmatic generation of scientific insight is still only in its beginning stages. As Turner et al. (2019) concluded about the empirical data situation (page 2810): “it is now abundantly clear that these in situ observations alone are not sufficient for unequivocally partitioning contemporary variations in atmospheric methane from 1980 to the present to specific source/sink pathways”. Regarding the atmospheric modeling efforts Lelieveld et al. (2016) observed (p 12488): “the differences between models are larger than between preindustrial, present and future emission scenarios calculated by the same models”.

This article highlights some of the most important empirical observations on methane that are yet to be satisfactorily explained and proposes avenues of how atmospheric modeling tools may need to be paradigmatically improved so that effective climate change mitigation strategies can be formulated. It is possible that with the improved paradigmatic understanding of the atmosphere, the assumption that a decrease in emissions will in a corresponding fashion lead to a decrease in atmospheric concentrations needs to be revised or refined. Instead, it is possible that atmospheric concentration levels are a function of non-linear equilibria, in which case it would become necessary to understand how these equilibria can be influenced in order for methane concentrations to decrease. In such a scenario, emissions reductions may be playing a different or less significant role than currently proposed.

This article is supported with Supplementary Material. Supplementary A describes where the methane is distributed in the atmosphere, and where it becomes destroyed. Supplementary B provides an overview of several of the controversies around describing and calculating the impact of atmospheric methane concentrations on climate change, including the “per-weight”, “multiplication factor” (Supplementary Figure S6), “potential”, “CO_2_ equivalency accounting” and the “feedback factor” controversies.

## The Most Important Empirical Observations Which a Model of Atmospheric Methane Needs to Explain

### The methane stagnation phase between 2000 and 2006 and the subsequent strong rise

**Figure 1.**
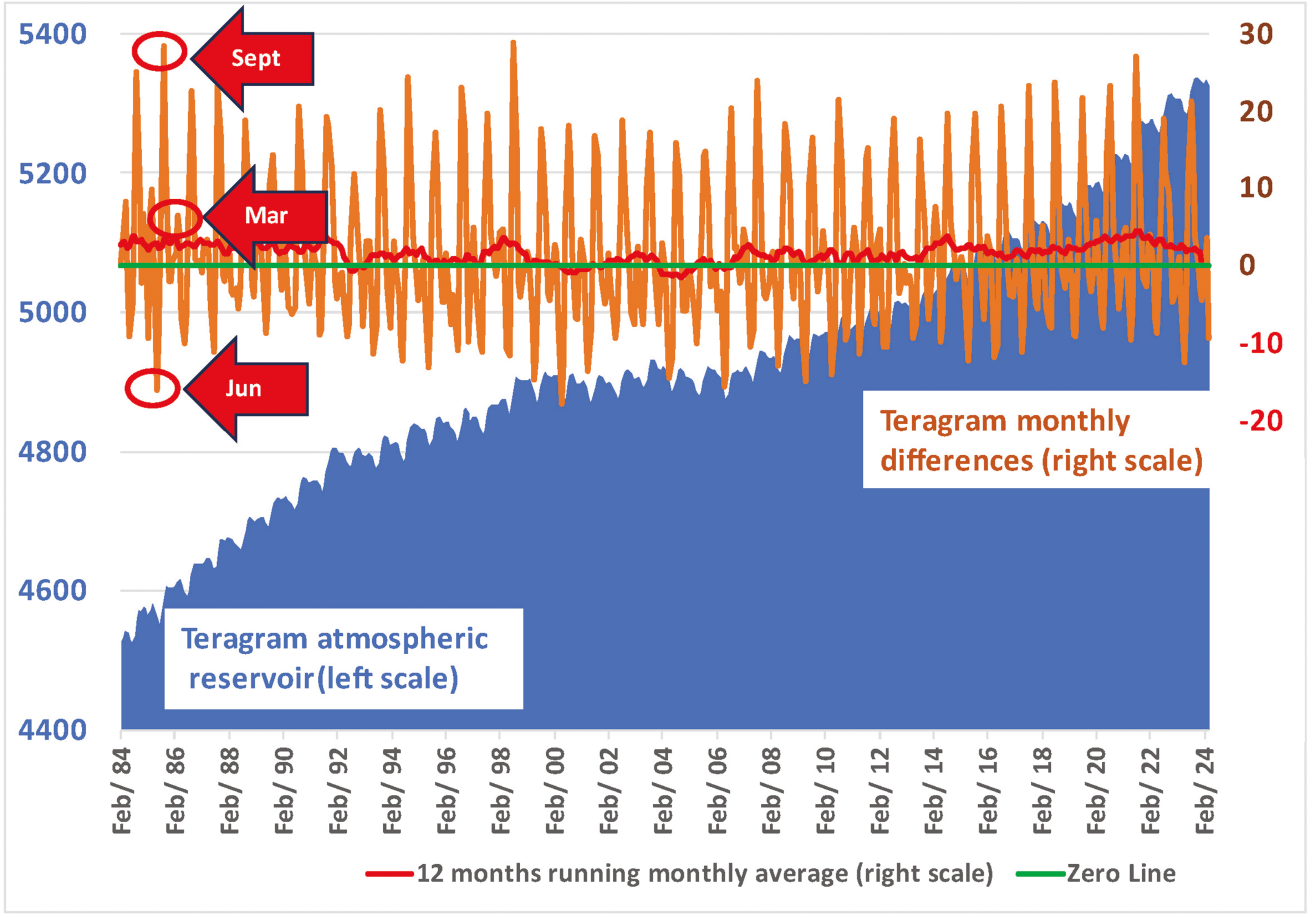
The dynamic of global methane reservoir between 1984 and 2024. GOALSciences.org illustration of data. The dark blue area is the size of the total atmospheric reservoir as it changed on a monthly basis between 1984 and 2024, which is the period of ground-based empirical measurements, with the unit teragram (one billion kg) on the left. The orange line is the monthly differences of that reservoir, with the unit teragram on the right. The reservoir always increases fastest in September corresponding to the boreal (northern hemisphere) summer, has a minor increase in March corresponding to the austral (southern hemisphere) summer, and shrinks the most in June (illustrated by red arrows). The red line shows the 12-month rolling average mean, also in teragram on the same right side scale. The blue area graph shows how the total methane reservoir did not increase between 2000 and 2006. Data are taken from the NOAA carbon tracker (Oh et al., 2024).

For as yet not satisfactorily explained reasons, the rise of atmospheric methane concentrations slowed since the mid-1980s, and then paused for 6 years, between 2000 and 2006 (Figure 1, blue area left scale). Thereafter, the rise resumed towards a record speed of increase, while there is no particular indication that methane emissions have increased correspondingly. Between 2007 and 2023, the concentrations increased by 140 ppb from 1780 ppb to 1920 ppb in just 16 years. For comparison, to put such rise into perspective: During what is considered to have been the previously fastest rise of North Atlantic region temperatures, the Bølling-Allerød warming period which ended the last ice age 14,700 years ago, methane levels increased by 140 ppb across 220 years. In that period global ocean water levels rose by 25 m, at a rate that is 20 times faster than today per year. In Greenland, temperatures rose by 10 °C, and across Europe and North America by around 5 °C (Naughton et al., 2023). In other words, to trigger a 140 ppb methane response would in the geological past require a far more drastic climate change event compared to today.

### The C13 isotope fractionation ratios

There also has clearly been a regime change in methane emissions in recent times, for which a resolutely convincing explanation is still outstanding. This is signaled by the so-called C13 isotope fractionation ratios. They result from the natural prevalence of the heavier isotope C13, which however is discriminated against by methane reactions (for more on the mechanics of the isotope fractionation ratios, see Supplementary A3 and Supplementary Figure S4).

**Figure 2.**
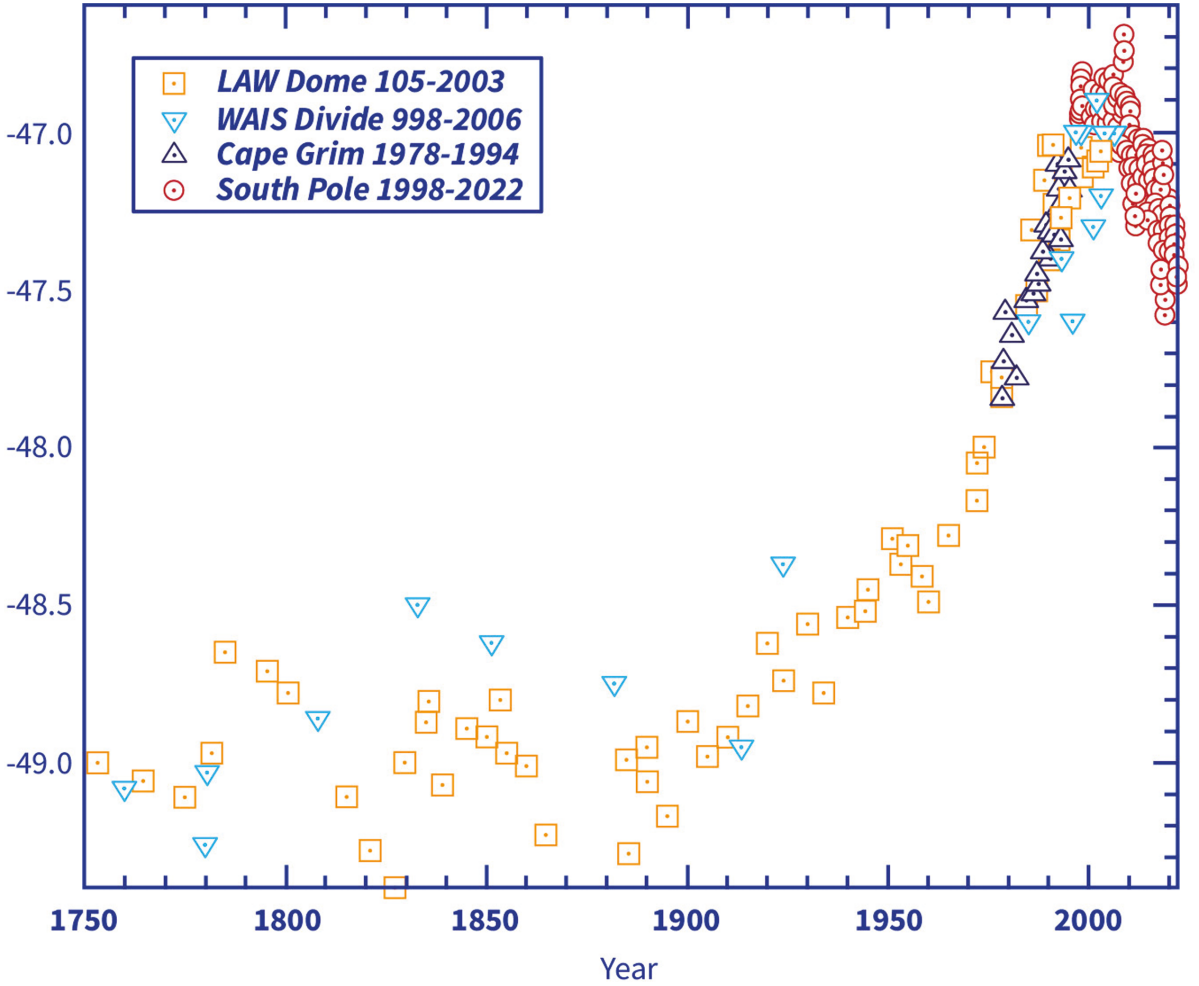
Two hundred and seventy years of methane C13 / C12 isotope fractionation ratios. Redrawn graphic from Nisbeth et al. (2023), their Figure 3. C13/C12 fractionation record from the South Pole since 1998, and ice core measurements since 1750, showing recent records in more detail. Data from Law Dome: Ferretti et al. (2005) and Rubino et al. (2019); and from NOAA (https://gml.noaa.gov/aftp/data/trace_gases/ch4c13/flask/surface/).”

While between the years 1900 and 2002, the isotope C13 fractionation values continued to go up (meaning the atmospheric methane mix became heavier with relatively higher portions of the isotope C13), since 2002, the fractionation values have been dropping rapidly (Figure 2). The background is that different emission sources and different destruction mechanisms of methane have different fractionation ratios, and therefore a change in the trend indicates that the composition of the sources over the past 20 years is much different from that of the previous 100 years, or the pattern of destruction is different, or both.

### The seasonal, subregional, and hourly variations

The CH_4_ atmospheric reservoir experiences monthly fluctuations. During the Northern Hemisphere, summer wetland emissions are strong, peaking in around July–August. One month later, in September, the winds will have carried these peak emissions to the oceanic recording stations of NOAA which are generally considered to be the main reference (Figure 1, orange line on right scale and red arrows). As the wetlands go into hibernation in autumn, the emissions go down and the reservoir will shrink again. A smaller uptick arrives in March, which corresponds to the January–February wetlands peak of the Southern Hemisphere. After that, with now both the North and the South in hibernation, the reservoir steeply dips to its lowest level in April–May, which is recorded by NOAA in June. During the stagnation phase from 2000 to 2006, the Northern summer emission peaks were neutralized by equally deep Northern winter emission dips (Figure 1, orange line). The 12-month moving average mean increase was almost zero at 0.07 Tg/month during those 7 years (Figure 1, red line). During the period from 2007 to 2014, the monthly mean went up to 1.21 Tg. In the period 2014 to 2021 monthly mean increased to 2.27 Tg, indicating an acceleration of methane concentrations (Figure 1, red line). Models can usually adequately represent these monthly fluctuations. However, methane concentrations also fluctuate greatly by hour or even minutes and seconds, and by meteorological conditions in subregions, as methane is mostly destroyed during sunshine hours (see Supplementary A 2, Figure S3). Those micro-dynamics are not captured by models as they are typically run in 3-hour or longer intervals. However, it is these micro-dynamics which could be indicative of the above-mentioned possibility of the atmosphere operating in different states of equilibrium with respectively higher or lower concentrations. By not accounting for these micro-dynamics, the current generation of atmospheric models are potentially overlooking a paradigmatic source of understanding.

### The 7-year cycle

**Figure 3.**
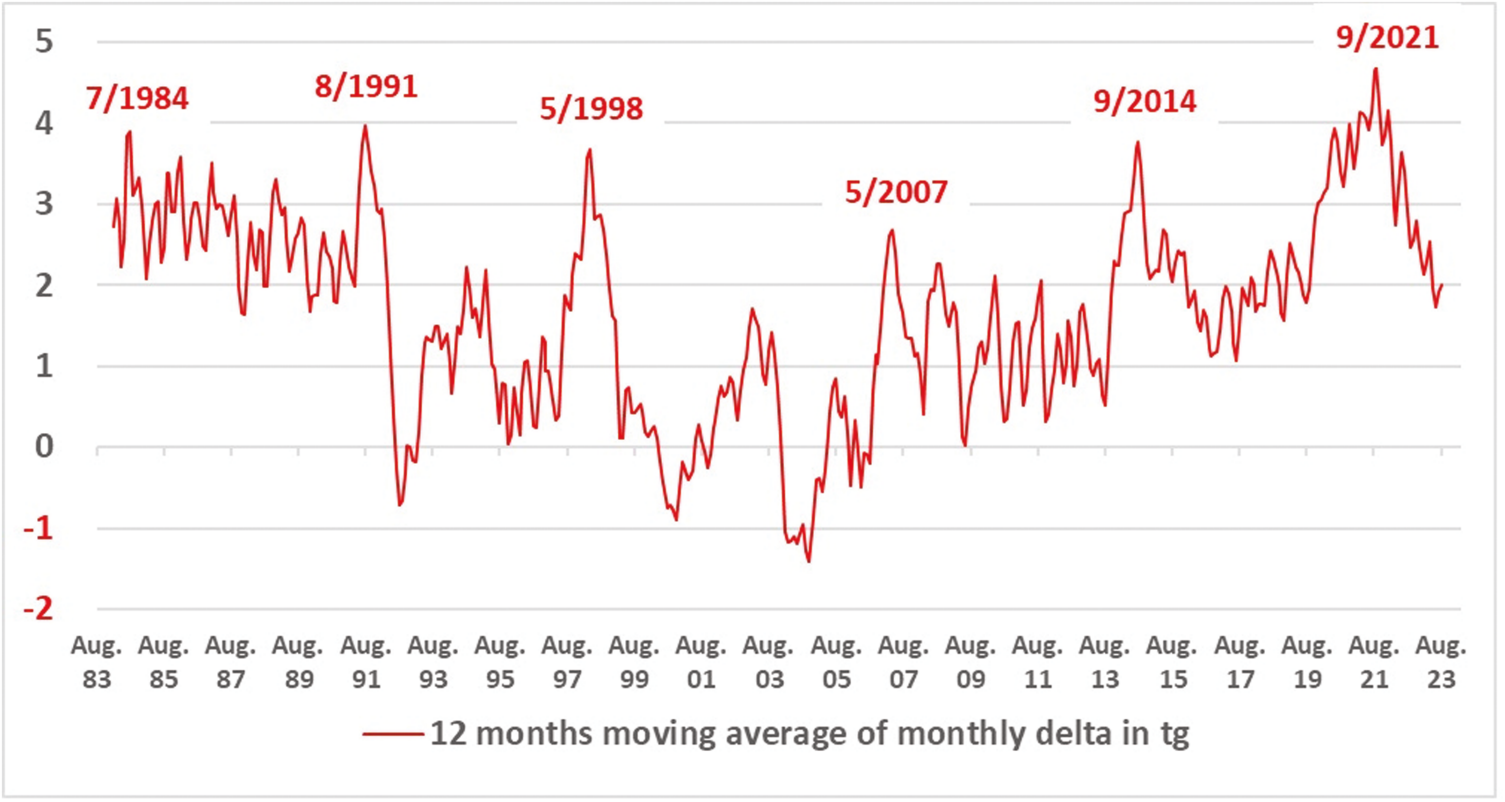
Two sets of 7-year cycles from 1983 to 2023. GOALSciences.org analysis of methane reservoir data. The 12-month rolling average of the monthly differences of the global methane reservoir displays two sets of peak years that are each 7 years apart. This line is the same as the red line in Figure 1, only at a different scale.

A 12-month moving average data analysis reveals a definite and robust but intermittent 7-year cycle of briefly rising methane concentrations in the atmosphere (Figure 3). To the best of our knowledge, this cycle has been noted just once in the literature so far by Khalil et al. (2007) and has since not been addressed. In personal communication with those authors, they confirm that they neither explained the cycle back then nor followed up on their publication. No further modeling efforts seem to attempt to explain the 7-year cycle. Direct highly resolved methane measurements are only available since 1983. For previous years the Greenland Cape Grim ice core measurements need to be used, which have a coarser chronology. Nonetheless, the 7-year cycle can be clearly discerned in the ice-core data reaching back to the 1850s and there are indications that they existed for the past 500 years as well (unpublished analyses by authors on ice-core measurement data, not shown here). Since there is no global meteorological phenomenon which exhibits such regularity, there must be a geological impulse which impacts the atmospheric methane concentrations. Explaining this geological impulse could also trigger a different paradigmatic understanding.

### An unprecedented increase in industrial times

**Figure 4.**
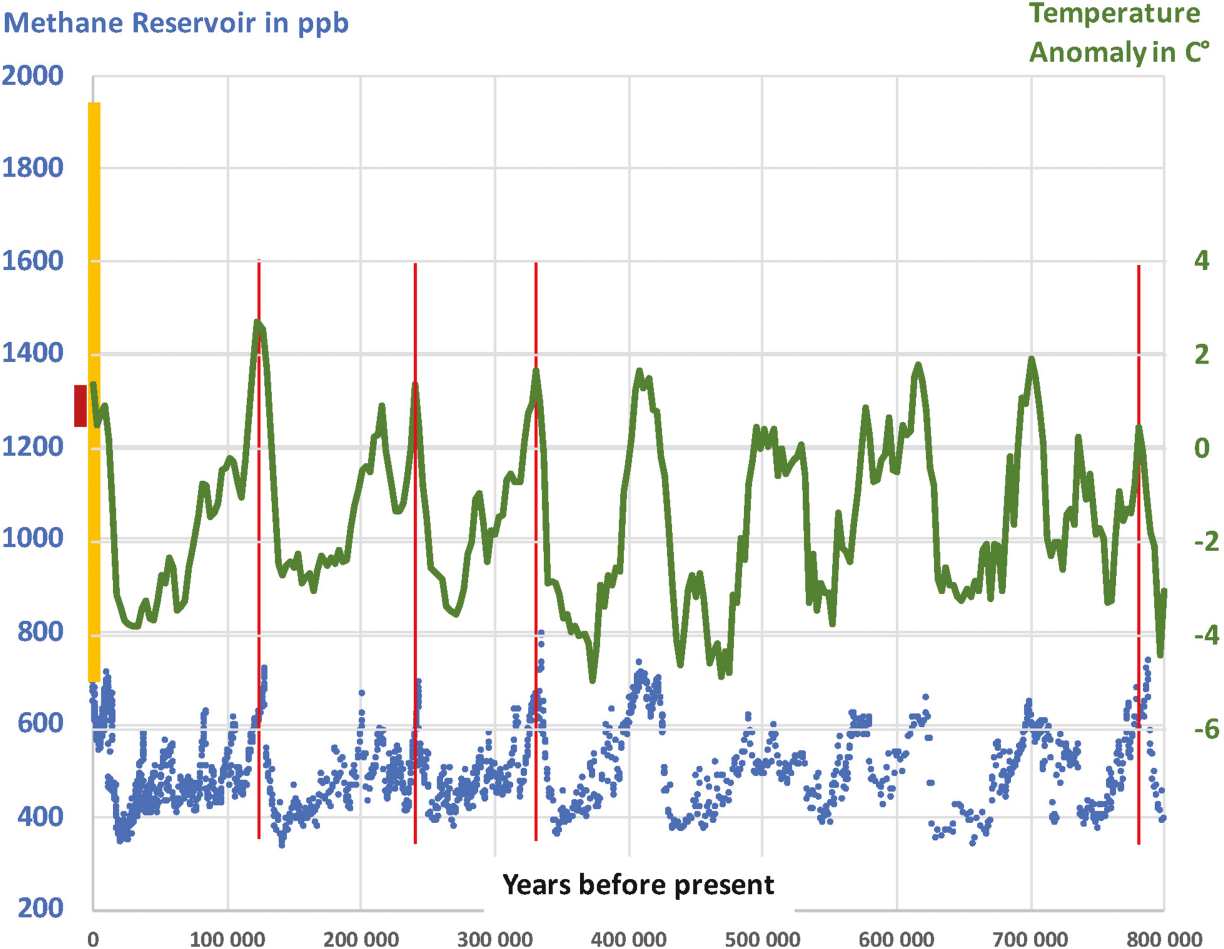
Of 800,000 years of methane emissions and temperature anomalies. GOALSciences.org illustration of data. The blue dots are methane reservoirs in ppb as measured by Antarctic ice cores (based on data by Loulergue et al, 2008). The green line is the global average surface temperature anomalies in C°—both over the past 800,000 years (based on data by Snyder, 2016). The thick yellow bar represents the increase of methane since the year 1750. The thick red bar represents the temperature increase since the year 1750. The thin red lines align methane and temperature peaks, showing that the methane precedes the temperature by several thousands of years, in most cases.

Methane concentrations have risen almost 3-fold throughout the industrial period, and at the same time to a level that is 2.5 times higher than any peak during the last 800,000 years (Figure 4), and most likely not seen since the age of the dinosaurs (Wilkinson et al., 2012). By comparison, the CO_2_ concentrations have risen by only about 51% during the industrial period. That drastic rise in itself above any paleolithic experience despite considerably less climate volatility during recent years compared to geological times, requires an explanation. The typically provided explanation is substantially higher human-caused emissions (for instance Saunois et al., 2024 and Supplementary A 1). However, that is not that clear. Then as of now, the majority of methane emissions were of biological origin. Then as now, there were a comparable number of methane-emitting ruminants deployed on global grasslands (Smith et al., 2016). Wetlands should have been emitting substantially more methane compared to today, because of their much larger extent. Large areas of what is today cropland in Europe, China, or the Americas were swampy wetlands until not too long ago. The extent of rainforests with their high methane emissions was much larger than that of today. Then as now, surplus non-eaten biomass (non-eaten by animals that is, such as excess fruits, nuts, seeds, dead grassy, or woody material) needs to be either fermented by bacteria or consumed by wild-fires, both of which emit methane. Then as now, geological emissions were tiny, at less than 5% of the total. The only real difference are the 20% anthropogenic fossil fuel methane emissions of today that did not exist before industrial times, but it is quite possible that they were compensated by the larger extent of wetlands and rain forests (see Supplementary A1, Figure S1 for current sources of all emissions). Global methane emissions for paleolithic times are generally assumed to be much lower than today, but that is only because the atmospheric models assume that methane lifetime is more or less fixed at currently observed levels—an assumption that is neither well proven nor plausible. Roughly speaking, if atmospheric lifetimes were assumed to be one-third of today in paleolithic times, then the same amount of emissions of today in those times, would lead to the one-third of concentrations during the paleolithic which we are observing. But then the research question would be an entirely different one to today. Researchers would then have to ask why lifetimes were so much different versus today, rather than whether or why emissions are higher. A thorough review and modeling of paleolithic methane chemistry is provided by Quiquet et al. (2015), (see also Supplementary B 11).

### The Young Dryas questions

12,800 years ago the dramatic Young Dryas occurred. Within just a few decades, North America and Europe saw a return of ice and ice age like temperatures that seemed to have disappeared during the previous warming. One thousand and two hundred years later, that ice disappeared as fast as it came. The mechanics of the Young Dryas are well understood to be the AMOC cycle (Atlantic Meridional Overturning Circulation, popularly known as the Gulf Stream), which turned off the heater in the North Atlantic, even if the precise course of events are still being studied (Cheng et al., 2020). While the climate effects of the Young Dryas were felt somewhat across the planet, it was primarily a North Atlantic event strongly affecting Europe and North America. The global sea levels kept on rising throughout that Young Dryas.

**Figure 5.**
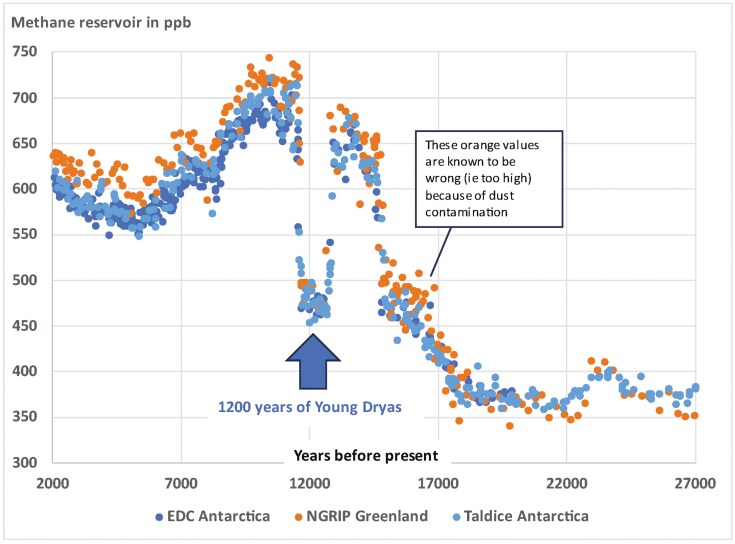
Methane fluctuations before, during, and after the Young Dryas period. GOALSciences.org illustration of data. Ice core measurements from two Antarctic sites and one Greenland site in comparison (based on data by Loulergue et al, 2008). Since the Young Dryas event, the Greenland methane concentrations have been about 8% higher than the Antarctic values, which is a difference that persists until today. The exceptionally fast drop and equally fast rise of the global methane concentrations are difficult to understand, as the Young Dryas was mostly a North Atlantic event.

Regarding methane, the Antarctica and Greenland ice core record (Figure 5) raises three questions for these end-of-the-ice-age years looking for an answer. First, during the Young Dryas period, global methane concentrations rapidly dropped back from 700 ppb to 500 ppb, stayed there for 1,200 years, and then rapidly ascended back to 750 ppb. Both the fall and the rise, are the steepest methane developments visible in the past 800,000 years (Figures 4 and 5), even though global average surface temperatures did not change much. This is remarkable as this is true for the global methane levels, even though the Young Dryas was a largely regional North Atlantic phenomenon. Second, until the Young Dryas, the difference between Antarctic methane levels and Greenland methane levels was most of the time negligible. Only during the brief peaks of the Dansgaard-Oeschger events during the glaciation between 60,000 and 25,000 ago, Greenland methane would typically be around 20% higher than in Antarctica (not shown in Figure 5, also Greenland ice core measurements only go back to 60,000 years, so we do not know about differences between hemispheres in earlier times). However, after the Young Dryas, that difference increased to 8% more methane in Greenland, where it remains stable until today (Figure 5). Third, from its peak right after the Young Dryas, methane levels began their typical rapid descent, just as in all previous instances of a warm interglacial since 800,000 years ago. However, at around 5,000 years ago, the trend stopped at 580 ppb (in the Northern Hemisphere), and then steadily kept rising until 730 ppb in CE 1750 (Figures 4 and 5). None of these three methane phenomena are well explained.

## Does Atmospheric Methane Modeling Require a Different Paradigmatic Approach To Be Able To Explain All Empirical Observations?

To be sure, there is no shortage of scientific articles that seek to explain the before-mentioned empirical observations. For instance, the currently most prominently published view is that the rise of emissions since 2006 and the turnaround in the C13 isotope fractionation ratios, would be due to a rise in wetland emissions in the tropics (for instance Lan et al., 2021; Yin et al., 2021; Basu et al., 2022; Oh et al., 2022; Zhu et al., 2022; Nisbeth et al., 2023). That at least seems to be the best way in which most of the currently designed models can be resolved with both constraints. In these publications, anecdotal evidence is subsequently sought whether the rates of inundation in the tropics have indeed increased during that period, which is difficult because satellites find it hard to penetrate cloud cover. However, these models cannot disprove that the same effect may come about if less methane were to be destroyed in the tropics, instead of more being emitted. This would then raise the question of why less might be destroyed, for which there is also no answer yet. Even more problematic is that if the modeled concentrations are so sensitive to wetland changes, then we would need to expect more methane volatility in paleolithic times, with its much larger temperature and water cycle swings—yet paleolithic volatility is lower than today. The argument could be that atmospheric chemistry was in a different “equilibrium state” in paleolithic times, which only raises the question: how is that equilibrium different?

The total methane reservoir by the end of 2023 had been 5,340 Tg, which had been rising by 46, 42, and 23 Tg during 2021, 2022, and 2023 respectively (NOAA Carbontracker Oh et al., 2024: https://gml.noaa.gov/webdata/ccgg/trends/ch4/ch4_mm_gl.txt, and Supplementary B 2) This reservoir is the result of four factors: a) the amounts of methane emitted into the atmosphere, b) the weather patterns by which the methane is distributed vertically and horizontally in the atmosphere, c) the spatial availability of the OH reaction partner with which the methane can react and thus becomes destroyed, d) delaying mechanisms in the atmosphere which prevent methane from being destroyed (see supplementary B 11, Figure S7 for model mechanics and Supplementary A1 and A2 for global methane emissions and destruction patterns, Supplementary Figures S 1 and S 2). The interaction between these four factors creates varying patterns of destruction mechanisms in the atmosphere including microvariations and non-linear effects, henceforth called dynamic micro-effects, which only become visible at the right size of the aperture of observation and modeling.

The difficulty with which the sciences are confronted is that none of these four factors are directly observable, and are therefore only poorly specified. The only variable that has become known in recent years in quite some spatial and temporal detail is the prevalence of the atmospheric CH_4_ reservoir that is the net result of these four factors. That is known by an increasingly dense measurement network of concentrations around the world comprising several hundred observation stations, as well as an increasing fleet of satellites that are looking at the concentrations from space (Supplementary A 4). From mostly that single outcome variable, everything else needs to be backward calculated. It is, in other words, an equation with many open variables, of which only one is well known.

Due to the increased interest in the role of methane in abating climate change, for about three decades there were so-called bottom-up and top-down approaches conducted to estimate where the observed methane concentrations originate from. The latest scientific consensus consolidation has just been published as a preprint and is under review (Saunois et al., 2024, as of November 2024). The bottom-up methods try to identify and estimate every single emission point, i.e., each cow, each pipeline, each rice field, etc, and add them up to a global total. As per Saunois et al., these bottom-up methods yield widely fluctuating estimates at plus/minus 14%, while the top-down methods fluctuate from plus to minus 3%. The top-down approaches use complex geochemical simulation models, called inverse models, which combine the typically prevailing weather pattern with the methane prevalence observations, and then attempt to track backward where the observed concentrations would likely have originated from. From that the models calculate the place, size, and type of emissions, and from that in turn the scale of methane destruction and the atmospheric lifetime. The models need to incorporate large datasets of empirical observations. The NOAA Global Monitoring Laboratory collects 25,000 datapoints at 60 stations annually. Each satellite collects millions of datapoints per day.

The computation run of such an inverse model can take several weeks even on modern supercomputers with several 100,000 cores. There are only a handful of computer models worldwide that can be operated for such top-down approaches. However, despite their complexity and sophistication, the inverse models still do not take into account the full spectrum of variability of dynamic micro-effects, because then the computational burden would become unmanageable. As a result, the confidence intervals for the outcomes tend to be broader than the rate of annual increases of the reservoir, and thus the models cannot resolve annual changes in the sources and the sinks of the methane reservoir, let alone seasonal, monthly or hourly changes. The general aspiration in the modeling community is that with more observations and more computing power, the confidence intervals can be reduced sufficiently, and thus more definite answers can be provided. Partially this is achieved by focusing on regional models and thus by reducing the computational load of limiting oneself to a smaller area, one is able to increase the spatial resolution. However, even the regional models quickly bump into the same constraints imposed by the dynamic micro-effects.

However, such hope of reducing the confidence intervals might be misplaced if the underlying paradigm for these models of a so-called “stiff” mechanistic system with linear, discrete, and independent chemical events is not appropriate. An alternative paradigm would be to think of the atmospheric methane reservoir instead as a non-linear system (NLS), which is a concept that has been gaining more traction in recent years (Selvam, 2010; Prather, 2021; Luo et al., 2023; Yang et al., 2024). At a sufficiently coarse resolution, an NLS can look analytically the same as a mechanistic system and can be analyzed with linear mathematics tools. However, at finer resolutions, non-linear effects can emerge such as solitons, chaos, singularities, or discontinuities, which leads to problems such as the fine grid cell dynamics do not add up to the coarse grid cell observations, and/or that large or sudden changes remain unexplained. The presence of NLS effects in the methane reservoir is well acknowledged as such, so much so that for instance sophisticated Kalman filters are used to filter them out and thus linearize the observations for the models. An alternative approach could be to instead of screening NLS effects out, focus the analysis on them as their essential feature.

Similar to other complex systems (for instance complex adaptive systems), NLS appears to be able to compensate for high levels of endogenous volatility among its elements giving them the appearance of high resilience, but at the same time can be vulnerable to relatively low impulses of exogenous volatility, upon which they might react with a surprisingly large degree of change. Under an NLS paradigm, fast-changing concentration levels of methane or reversals of fractionation ratios or periodic cycles, would not be considered to be the result of a deterministic change in the rate of emissions or destructions as they are currently treated by the typical inverse models, but would be the result of an NLS adjusting to a different equilibrium state in response to an exogenous shock. Such a paradigm would require different analytical tools than those that are currently deployed in atmospheric methane modeling. Given that the current fleet of models struggles to explain the empirically observed dynamic micro-effects, and even cannot explain some of the large-scale effects as shown in the first section, maybe the time has come for a different paradigmatic approach.

## Conclusion

Current modeling techniques for atmospheric methane, as sophisticated as they are, fail to satisfactorily explain the record of empirical observations for both modern and geological times. Several phenomena are not unambiguously explained, including both the 2000–2006 plateau and the subsequent fast rise, the reversal of the C13 fractionation ratios since the early 2000s, or a mysterious intermittent 7-year cycle that has likely existed for centuries. Another puzzle is why methane can rise so fast in modern times during a period of minimal climate volatility compared to much more volatile paleolithic times with a lot more subdued methane fluctuation. Both the range of terrestrial and satellite-based observation systems has increased tremendously in the last few years. Whereas in the 2010’s there was only one space-based observation instrument available for methane (Sciamachy, https://www.esa.int/Applications/Observing_the_Earth/Envisat/Envisat_enables_first_global_check_of_regional_methane_emissions), nowadays there are almost 20 (Supplementary A 4, Figure S 5). However, increased observation density does not help if the models are paradigmatically inadequate. In particular, the paradigm in the models of attempting to linearize non-linear micro-dynamics seems particularly prone to revision. In short—the quest for conceptually improved methane models that can account for all empirical observations is still open.

## Supplementary Data

Supplementary data are available at *Animal Frontiers* online.

vfaf001_suppl_Supplementary_Figures

## About the Author(s)

**Peer** **Ederer** is the founder and director of GOALSciences, the Global Observatory of Accurate Livestock Sciences, which has the mission to research and communicate scientific evidence about the role of animals in the global food system. He has an MBA degree from Harvard University, a PhD in financial economics, and holds an adjunct professorship for innovation studies. He has extensive experience in strategy advisory to private companies and public bodies in the global food system and is a frequent presenter on related topics. He has been engaged in scientific research in cooperation with globally leading universities from around the world and is a member of the Scientific Council of the World Farmers Organization. **Corresponding author**: peer.ederer@goalsciences.org

**Taras Iliushyk** received his master’s degree in computer science, specializing in computer technology in Biology and Medicine at the National Technical University of Ukraine «Igor 392 Sikorsky Kiev Polytechnic Institute». Since then, he has deepened his knowledge of coding in different software languages, with a special emphasis on creating data science applications for the processing of big data sets.
