# Supplementary material for: In search of better models for explaining atmospheric methane accumulation
Source: Anim Front. 2025 Apr 5;15(1):34–42. doi: 10.1093/af/vfaf001 (PMC11971521; doi:10.1093/af/vfaf001)
Supplement: vfaf001_suppl_Supplementary_Figures [file vfaf001_suppl_supplementary_figures.pdf]

In search of better models for explaining atmospheric methane accumulation

In Animal Frontiers, Vol 15, Issue 1

Supplementary Documentation

Authors:

Peer Ederer * [a)], Taras Ilyushik [a)]

a) Global Observatory for Accurate Livestock Sciences, Switzerland

* Corresponding author: peer.ederer@goalsciences.org

Content

Supplement A 1 – 4

Where is the atmospheric methane, how much is emitted where and by what, and where is it destroyed, what are isotope fractionation ratios and which satellites are observing methane?

Supplement B 1 – 12

Overview of some of the controversies around describing and calculating the impact of methane concentrations on climate change, starting with some definitions.

The references include those of the main text of the Animal Frontiers Issue

Supplement A) Where is the atmospheric methane, how much is emitted where and by what, where is it destroyed, what are isotope fractionation ratios and which satellites are observing methane?

A 1. Presence and emissions of methane

The average atmospheric volume mixing ratio of marine surface methane (CH_4) in 2023 was 1922 parts per billion of air molecules (ppb), according to US National Oceanic and Atmospheric Administration (NOAA) (https://gml.noaa.gov/webdata/ccgg/trends/ch4/ch4_mm_gl.txt) (Oh et al, 2024). Generally speaking, the Northern Hemisphere north of 12° latitude (NH) has a 8% higher mixing ratio than the Southern Hemisphere south of 12° (SH). The tropics between N 12° and S 12° have a 5% lower mixing ratio than the NH. Hourly dynamics aside (see below), the mixing ratios are fairly uniform up until the upper middle levels of the troposphere. In the upper levels of the troposphere and the lower levels of the stratosphere they are about 20% lower. Methane mixing ratios towards the upper levels of the stratosphere can be as low as only 100 ppb, or only 5% of Earth's surface levels. (The concentrations are much lower in higher altitudes, because of lower air density, but here the unit is mixing ratio, see below in Supplement B 1 for more explanation).

Figure S 1 shows in which latitude and longitude area how much of which methane source is emitted. CH_4 is emitted by biological, physical and geological sources on or below the Earth's surface. CH_4 has no atmospheric source, therefore all CH_4 in the atmosphere is from terrestrial origin. The biological sources derive from microbes which digest or catalyze biomass material under anaerobic conditions which results in CH_4 creation and emission (Conrad, 2020). This occurs for instance in wetlands, rice fields, enteric digestion in ruminants and manure, in other wildlife such as termites or in wastewater treatment. Methane which is produced in wetlands might either be emitted through respiration mechanisms of plants or be at first transported via water streams. Another only vaguely understood biological process might be the emission of CH_4 by all living organisms under oxic conditions, which could only be ascertained to be occurring in 2022 (Ernst et al 2022, Mao et al 2024), but which extent is still under investigation.

According to the latest global methane budget estimations for the period between 2010 and 2019, the global consortium of scientist's consensus estimate of annual emissions split of the annual total of 573 tg (middle top down estimate between 553 and 586 tg, Saunois et al., 2024):

- Fossil fuels (physical: coal mining, oil & gas, industry, transport, residential): 115 tg
- Ruminant's enteric fermentation and manure (biological): 112 tg
- Rice fields (biological): 32 tg
- Anthropogenic landfills and waste (biological): 84 tg
- Anthropogenic biomass and biofuel burning (biological): 27 tg
- Wetlands and fresh water (biological): 165 tg
- Wild animals and termites (biological): 12 tg
- Geological (volcanoes, oceans, according to Petrenko 2017): 15 tg
- Other geological (permafrost soils, wildfires): 14 tg

(tg = teragram = 1 million tons = 1 billion kilograms = 10^{12} grams)

58% of the global methane emissions are emitted in the Northern Hemisphere north of 14°, 9% south of 14°, and 33% in the tropical zones between N 14° and S 14°.

Figure S 1: Annual emissions of methane by longitude, latitude and source

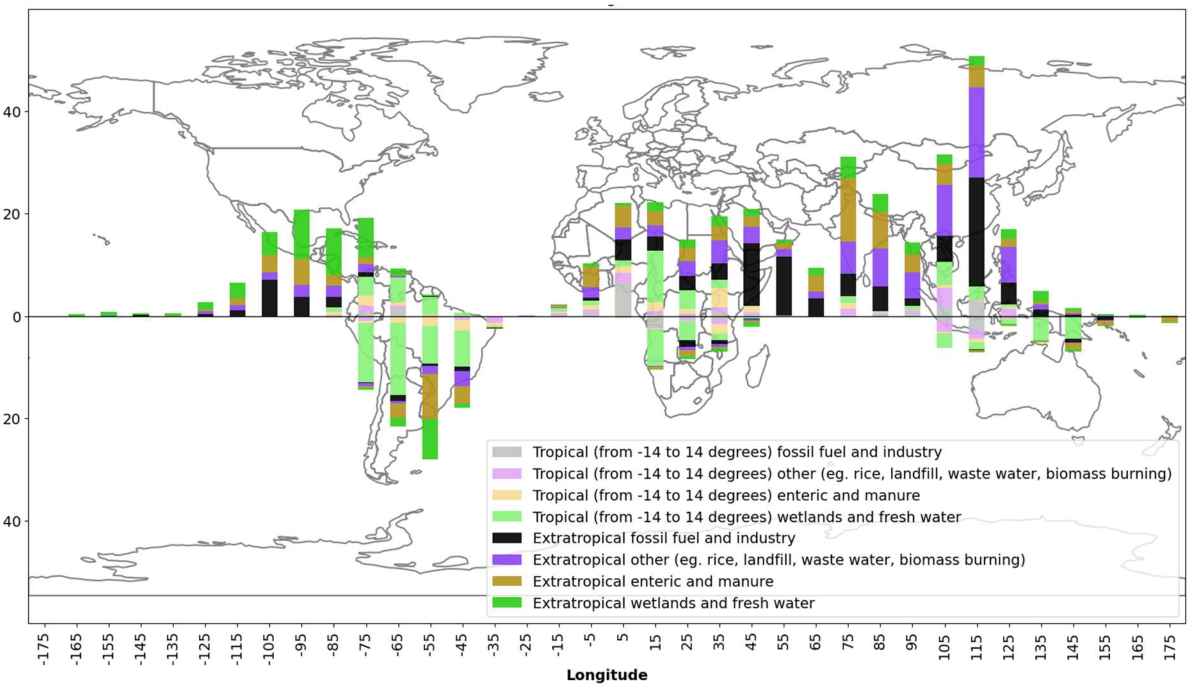

GOALSciences.org illustration and analysis of data. All data except wetlands are sourced from EDGAR https://edgar.jrc.ec.europa.eu/dataset_ghg80. The wetlands and fresh water emissions are modelled with wetCHARTs version 1.0 (Bloom et al., 2017). The small amounts of geological sources are not included because they cannot be assigned to a longitude location. The unit for the bars is annual teragram of emissions.

A 2. Destruction zones and timing of methane

The main destruction area of methane occurs in the tropical belt, because the main destruction reactant hydroxyl OH is formed under conditions of intensive sunlight and presence of water vapor. Around 70% of all global methane is destroyed in the tropical and near-tropical zone of N 24° to S 24° latitudes. According to Saunois et al. (2024), 93% of atmospheric methane is destroyed by OH, 1% by chlorine, and 6% is taken up by soils. Other researchers estimate that chlorine might account for as much as 3% of total destruction, if ocean spray chemistry is also considered (Herpen et al., 2023, there also more references). Case studies of highly polluted urban centers suggest an important role for chlorine destruction of methane there (Soni et al., 2023). Some investigations have shown that cave atmospheres destroy methane highly effectively, and that this might extend to the uppermost vadose zone of subterranean atmosphere even without intervention of methanotrophic bacteria (Fernandez-Cortes et al., 2015). However, whether that is a factor that can be seen as included in the above-mentioned 6% soil uptake, or is additional, is uncertain. Regardless of these detailed nuances, the overwhelming destruction method of methane is by oxidization with the OH radical. Figure S 2 illustrates in which longitude and latitude regions the methane is destroyed. In the tropics, due to rapid methane destruction, the methane mixing ratios change on an hourly basis, as is illustrated and explained by Figure S 3.

Figure S 2: Annual destruction rates of methane by longitude and latitude

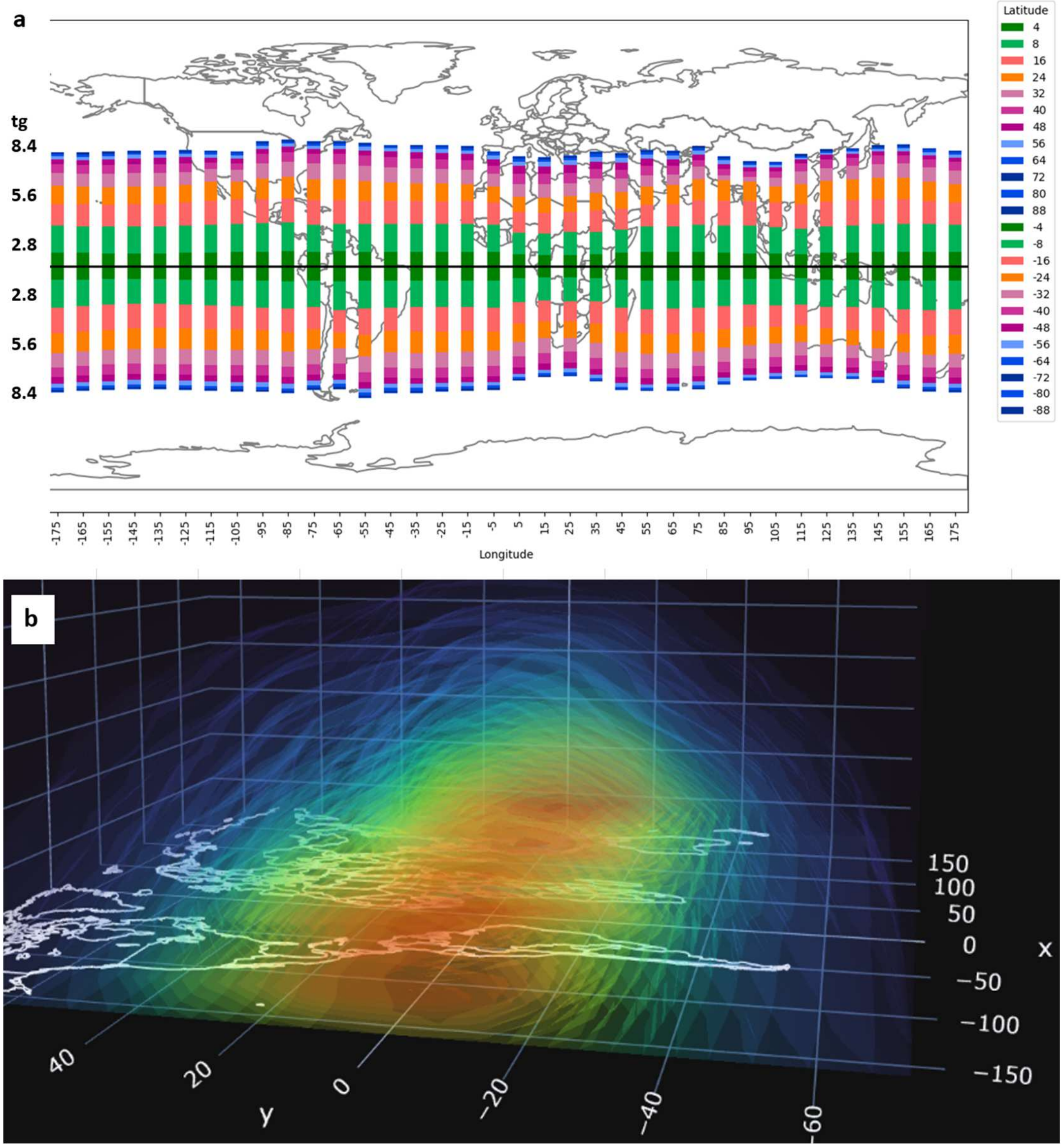

GOALSciences.org illustration and analysis of data. 2D and 3D rendering of planetary zones where methane is destroyed for the year 2019; a) In the 2D picture each color bar represents the amount of methane destroyed in a 10° longitude band, for each 4° latitude, in teragram (y-axis is tg for the bars, not ° latitude). The values are based on author's calculation of one-box cells coupled with a global atmospheric chemistry model (respectively Caaba by German Max Planck Institute, Sanders et al. (2019), and GEOSChem 14.2. Bey et al. (2021) and Zhuang et al. (2019)). To obtain these values, we ran 108,339 one-box cells coupled with the inversion system, wherein each one-box cell calculates 3384 different chemical reactions in five minute steps for a 5 day duration; b) shows the same values as a screen-shot from an interactive 3D rendering of the data.

Figure S 3: Daily and hourly cycle of methane mixing ratio in global atmosphere

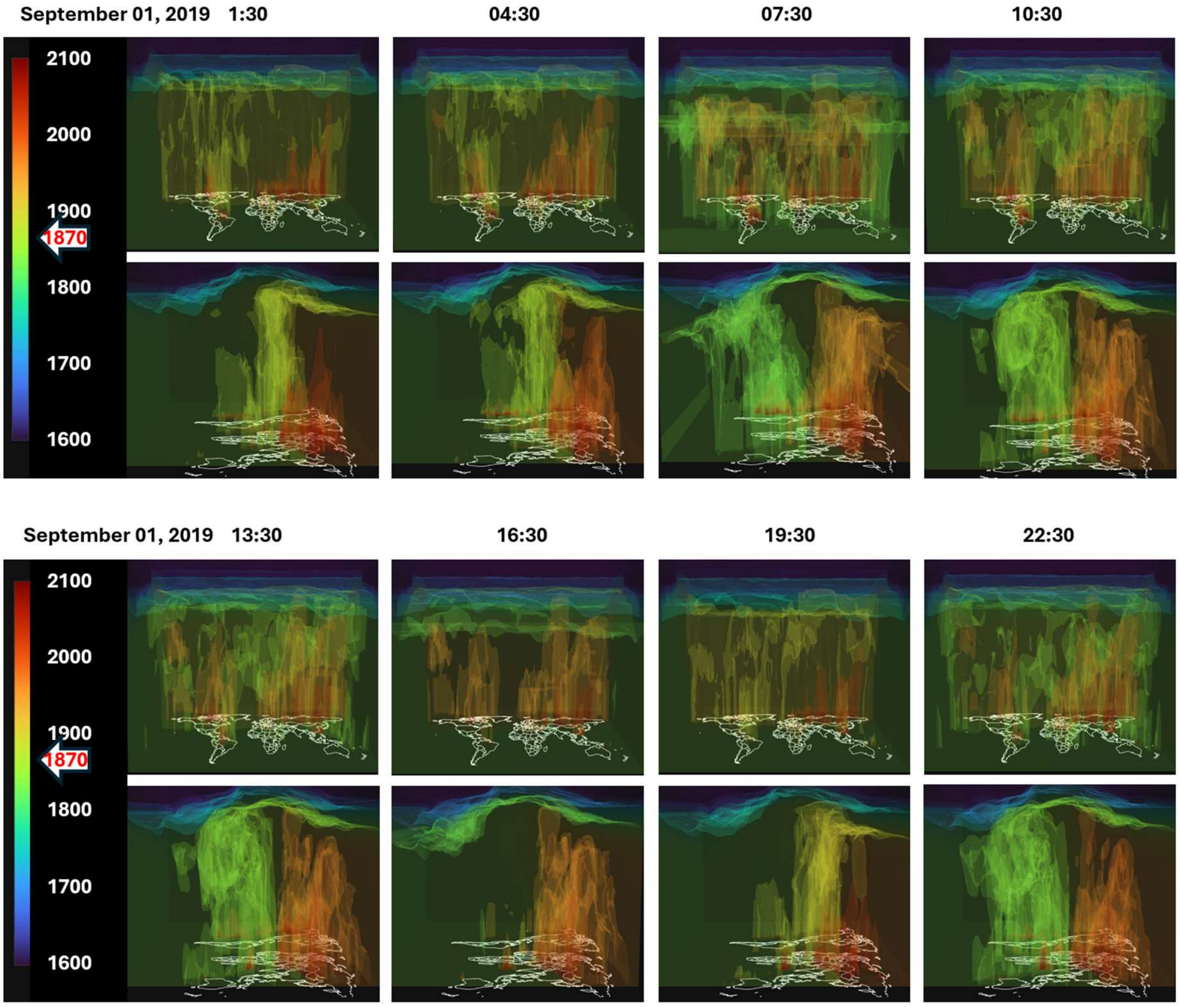

GOALSciences.org illustration of data. Still frames of interactive 3D rendering of atmospheric model output data from the NOAA Carbontracker team (data available here: https://gml.noaa.gov/ccgg/carbontracker-ch4/download.html. (Oh et al., 2024). Each hourly rendering contains 2.16 million datapoints. Shown here is the example of the 1st September 2019 for its pronounced meteorology. The unit is ppb atmospheric mixing ratio. The red value of 1870 ppb on the left legend, marks the global average for September 2019. Accordingly, all green and blue "bedsheet" colors indicate lower than average mixing ratios, and brown and reddish tones indicate regions with higher than average mixing ratios. The times are synthetic, so for instance the 01:30 panel displays the data as if it is 01:30 in the morning everywhere in the world at the same time. Upper panels are looking from South to North, and lower panels are looking from East to West. The map of the world is projected for orientation.

The lower panel at 01:30 shows the typical above average of methane prevalence in the Northern Hemisphere (brownish colors), and lower than average methane prevalence above the tropics and the Southern Hemisphere (greenish colors). At the highest altitudes, the troposphere has much reduced levels of methane (blueish colors).

On this particular day of September 1st 2019, the typical dry season high pressure systems above the Southern tropical seas were operating. The light green fields at 07:30 indicate how the methane-poor air from higher altitudes sinks above the tropical ocean and Southern regions. At 10:30 the air currents reverse. The tropical convection sets in, drafting surface air upwards towards the tropopause and there polewards into the Hadley Cells, visible by the swirling motion at the upper end. This ascending air has

lower than average methane, because rapid methane destruction is going on during the day, as OH production is at its maximum. By 16:30 the convection has stopped and the air has completely mixed up, so that fewer “bedsheets” of different concentrations are visible. Due to the methane-enriched air that reached it during the day, the tropopause has now its highest methane readings (shown by light greenish instead of blueish). By 22:30, the night time sinks above the land, taking with it the methane-poor air from above.

A 3. The C13 isotope fractionation ratios of methane

Isotope fractionation ratios (or factors) are an important tool to identify sources and regions of methane emissions in atmospheric modelling. The ratios can help to co-determine the models better. However, there are limits to this approach, due to the complex pattern of chemical reactions involved (figure S 4).

About 1.1% of all carbon in the atmosphere are constituted with the heavier carbon isotope C13 instead of the regular C12 (C13 has seven neutrons instead of the common six). Chemical and biological processes do not react with these C isotopes in proportional shares. Biogenic processes like photosynthesis prefer to react with the lighter C12 molecule in the atmospheric CO2, and therefore their bound carbon is lighter than the atmosphere (they have a higher fractionation ratio = a more negative number = less C13). However, C3 and C4 vegetation have differing degrees of preference, with C3 plants preferring more C12 than C4 plants (C3 plants are typically trees, grains and cold climate species while C4 plants are grasses, maize, sugar cane and generally plants in the tropics and warmer climates). So C3 plants have a higher fractionation ratio (factor) than C4 plants. Microbial digestion of biomaterials also prefers the lighter isotope of C12, so that its resultant methane emissions will be even lighter (ie have an even higher fractionation ratio = a more negative number = even less C13). Fractionation ratio results of enteric fermentation thereby depends also on which fodder (C3 or C4 biomass) the ruminants have eaten. Biomass which does not get microbially digested, but burnt, leads to heavier methane with less negative fractionation values because it does not pass through the discriminate bacteria.

The chemical reactions also discriminate. Methane reactions with OH discriminate against C13 only a little bit, so that the left behind (non-reacted) methane is only a little bit heavier than before (low fractionation ratio in destruction = > negative value is made only slightly more positive = left behind methane has slightly more C13). Methane reactions with chlorine

discriminate much against C13, so that it has a higher fractionation ratio, meaning it will make the remaining substance much heavier = a lot less negative = more positive = more C13. A heavily C13 depleted methane substance measured in the atmosphere (a strongly negative number with much C13 missing compared to the baseline), could therefore be the result of either more methane production from biological fermentation of C3 vegetation (fermentation being more negative, and C 3 being more negative), or of methane destruction by mostly OH (only slight positive effect), and not chlorine (strong positive effect).

Fossil fuel sources of methane can have a wide-ranging profile of C13 content, ranging from heavily depleted to only lightly depleted, depending on which oil or coal field they derive from, further complicating the picture.

Figure S 4: Overview of fractionation ratio impacts by sources and reactants

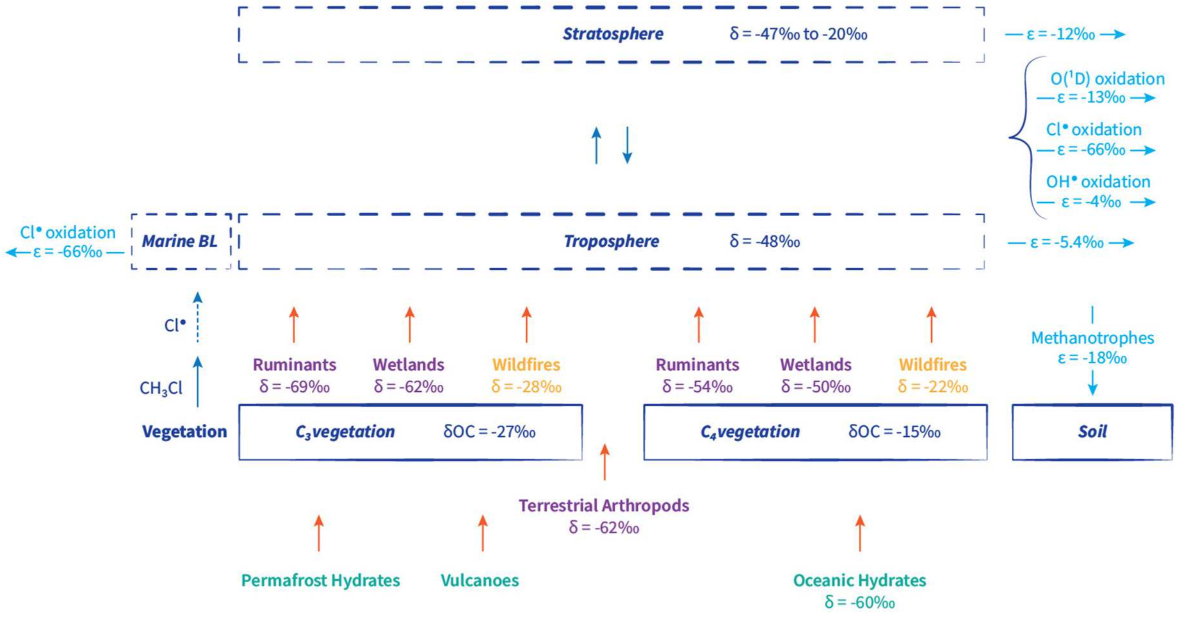

Redrawn and corrected graphic from figure 2.3 of Haller 2013. Methane budget with corresponding isotopic signatures and effects on fractionation ratios, showing sources of methane to the atmosphere in purple, yellow and green, and sinks in light blue.

A 4. Satellite observations of methane

Figure S 5: Space-bound observation satellites as per 2022 (more satellites are on their way)

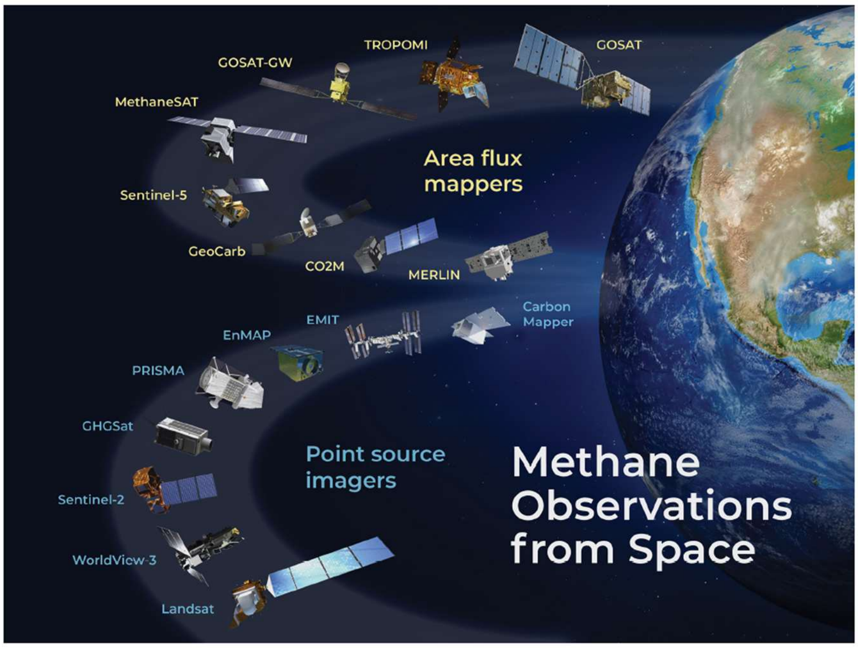

Source: https://acp.copernicus.org/articles/22/9617/2022/

(copyright license CC BY 4.0 granted)

Launch dates of major methane observation satellites

GOSAT	2009	Japan Aerospace Exploration Agency
Landsat-8	2013	US Geological Survey
WorldView-3	2014	Maxar
Sentinel-2	2015	European Space Agency
GHGSat*	2016	GHGSat Inc.
TROPOMI	2017	European Space Agency
Gaofen 5	2018	China High-resolution Earth Observation
PRISMA	2019	Agenzia Spaziale Italiana
EnMap	2022	German Aerospace Center (DLR)
EMIT	2022	NASA
GHOSt**	2023	Orbital Sidekick
MethaneSAT	2024	Environmental Defense Fund
Carbon Mapper**	2024	Carbon Mapper, RMI, NASA-JPL, CARB, Planet, others

*Currently a 12-satellite constellation. **Currently a three-satellite constellation. ***Due for launch in 2024, initially as a two-satellite constellation with more to follow in future years.

Source: https://rmi.org/methane-satellites-101-more-eyes-take-to-the-skies/

Supplement B) Overview of some of the controversies around describing and calculating the impact of methane concentrations on climate change, starting with some definitions.

B 1. What is a mixing ratio?

A mixing ratio is similar to concentration, however the former has the unit of parts per air molecules and the latter parts per volume such as m^3. For most purposes, the terms can be used interchangeably. Since different molecules have different weight, and since the concentration changes with the amount of air pressure, whereas for climate warming discussion what matters is the share of methane among all other molecules, it is common in atmospheric chemistry to use the term mixing ratio and its corresponding unit of parts per billion (ppb).

B 2. How much methane is in the atmosphere?

The 2023 surface mixing ratio of methane was 1922 ppb (or 1.9 pp million). Taking into account the progressive change of the mixing ratio towards upper atmospheric levels (Supplement A 1), this translates into a total of about 5340 million tons (or 5340 teragram = tg) of methane in the atmosphere (Lan et al. (2021) for conversion factor of 2.763 in her supplement equation 1), which is interchangeably called either atmospheric methane burden or methane reservoir. The preindustrial level had been slowly but steadily rising for about 5000 years from around 570 ppb. In the previous 800,000 years it had fluctuated between 350 and 800 ppb, with an approximate median at around 520 ppb (well-known from highly resolved ice core sampling in Antarctica, figure 4 in main text).

B 3. What is the radiative efficiency of CH4 versus CO2 ?

CH_4 molecules have a 29 times higher radiative efficiency than carbon dioxide (CH_4: 0.000388, CO_2: 0.0000133 W m^{-2} ppb^{-1}; IPCC AR 6, Chapter 7 SM, Table 7.SM.7, p.16). At the same time, CO_2 is 218 times more prevalent in the tropospheric portion of the atmosphere (in December 2023: CO_2: 421 ppm, CH_4: 1.9 ppm, NOAA Carbontracker https://gml.noaa.gov/ccgg/trends/). In practical terms. this means that in any given volume of average tropospheric air in the year 2023, and not adjusted for other effects (see below), the contained CO_2 would have contributed 7.5 times more potential radiative forcing to atmospheric warming than the contained CH_4 (218/29 = 7.5, or 13%). In other words, a priori, CH_4 is almost one magnitude less important to atmospheric warming than CO_2.

B 4. Computations for "methane is 28 times more climate warming effective as CO2"

In popular discourse, and also too often in scientific or peri-scientific literature it is frequently written that CH_4 would be 28 times more climate warming effective as CO_2, rather than the opposite 7.5 times less than CO_2 mentioned above. However, there are several misunderstandings and misconceptions associated with this number 28.

This value 28 was calculated for the IPCC AR5 report, published in 2013. This value did not include climate feedback. The value which included climate feedback was 34 (IPCC AR 5, Chapter 8, Table 8.7, page 714). In the IPCC AR6 report, published in 2021, the IPCC texts did not provide a value without climate feedback anymore, which makes them look as if the values remained almost the same, while actually they were substantially revised downwards compared to AR5. Furthermore, AR6 now (correctly) provides two values for fossil-origin and non-fossil origin, which are respectively 29.8 and 27.0 (IPCC AR6, Chapter 7, Table 7.15, page 1017). The difference accounts for the fact that methane degrades in the atmosphere into CO_2 and water. In case of the fossil origin, this CO_2 represents a net addition of CO2 into the atmosphere because its "C" was retrieved from carbon stocks below Earth's surface, whose climate warming effect needs to be included. For non-fossil origin methane, the "C" atom had been previously captured from atmospheric CO_2 by photosynthesis, and is therefore no net addition to the carbon stock in the atmosphere. (In AR4 from 2007, the value was 25 for feedback not included, which is also a number that keeps on circulating).

These values, whether 25, 28, 34, 27.0 or 29.8, refer to the equivalent Global Warming Potential (GWP) of methane *by weight* over a 100-year time span compared to CO_2, and are therefore referred to as GWP100. The resulting CO_2 values are called CO_2eq. The AR6 GWP values by weight over a 20-year time span are respectively GWP20 82.5 and 79.7.

However, this IPCC calculation involves several controversial considerations and assumptions, which will be further outlined below.

B 5. The "per-weight" controversy

CO_2 is 2.74 times heavier than CH_4, and thus on a molecule per molecule basis rather than gram per gram as in the IPCC calculation, the GWP CO_2-equivalency of biogenic methane is only a factor 10 instead of 27 (27 / 2.74 = 9.85) (Flood 2011). There is no particular reason why a per weight comparison should be preferred over a per molecule comparison, especially since usually the per molecule comparison is utilized (for instance with parts per billion mixing ratio description). A recent prominent example of this mischaracterization is the introductory chapter by Saunois et al 2024, where a large consortium of scientists have been prominently compiling the most recent global methane budget effort: *"For a 100-yr time horizon and without considering climate feedbacks the GWP of CH_4-fossil is 29.8 (CH_4-non fossil GWP is 27), whereas the values reach 82.5 over a 20-year horizon for CH_4-fossil and 79.7 for CH_4-non fossil (Forster et al., 2021)."* It is not mentioned in this introduction to the Saunois et al article that these GWP values refer to a comparison by weight, and it is also not correct that the values are not considering climate feedbacks, because they do. It is also not clear why Saunois et al mentions the 29.8 factor for CH_4 fossil first, and for non-fossil in brackets, when they will be fully aware that 78% of all emissions are of biogenic origin (see Supplement A 1), (their publication is still under review and in preprint status, and thus these errors might yet be corrected before final publication).

B 6. The "multiplication factor" controversy

Methane's radiative efficiency potential in the atmosphere is due to it being excited by the earth-surface reflected infrared spectral wave bands centered around 1.65 µm, ~3.3 µm and ~7.7 µm (Nelson et al 1948, Wu et al 2024) and thus methane captures this photonic energy instead of it being released straight back into outer space (figure S 6). This is called the abundance-based radiative forcing. This forcing is multiplied with a factor to become the emissions-based radiative forcing, because methane also causes indirect warming effects in the atmosphere. For instance, when it is destroyed, some of the resulting products are ozone or water, both of which are also greenhouse gases. In AR4 from the year 2007, these multipliers were 25% for ozone and 15% for water vapor generation in the stratosphere. In AR5 from the year 2013, these factors were increased to 50% and 15% respectively, for a total of 65% multiplication factor (IPCC AR 5 WG 1 Chapter 08 Supplementing Material page 17).

Figure S 6: Spectral absorption bands of methane and other GHG gases

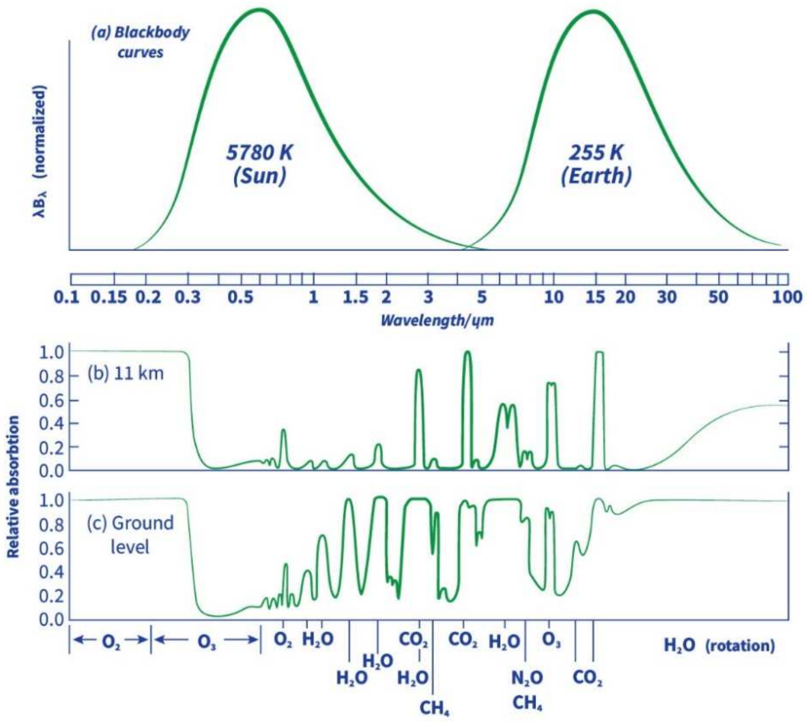

Redrawn graphic from Haller 2013, his figure 2.1 on showing the spectral absorption bands of the various greenhouse gases including CH4.

In AR 6 from 2021 the multiplier appears to be revised upward to become factor 100% (IPCC AR 6 Chapter 6, 6.4.2 page 855 summary), which was rounded down from a best estimate of 121% as per Chapter table 6 SM1 (IPCC AR 6, Chapter 6, Supplementary Material, page 3, value for GMERF of 1.195 W/m^2) versus Chapter 7 table 7.8 (IPCC AR 6, Chapter 7, Table 7.8, page 960, value for GMERF of 0.54 W/m^2, which in itself has also increased from 0.48 W/m^2 compared to AR 3, 4 and 5). In AR6, the multiplier factor for ozone appears to have gone down to 32%, and the multiplier for stratospheric water went down to 6%, but it is not clearly mentioned in the IPCC texts. However, before these multipliers are applied, the methane value is first subjected to a new multiplication factor of 56% in order to account for air chemistry effects with NO_x and VOCs – which when all is multiplied in the end, gives methane a factor 1.21 higher radiative forcing, than it has alone by itself. Commenting on all these variations, the IPCC text merely says that *“direct comparison of results with AR 5 are difficult”* (IPCC AR 6 Chapter 6, 6.4.2 page 853), leaving the reader alone with trying to reconcile and replicate the changes.

It needs to be emphasized that all of this is just modeling, and is only faintly based on empirical observation. The models incorporate numerous complex assumptions, that are not made transparent in the IPCC reports. For instance, though it is claimed that the IPCC AR6 methane modelling includes short-wave near infrared absorption (eg Allen et al., 2023 - which would lead to a lower greenhouse gas effect), it is then written that other models have found unspecified but additional greenhouse gas strengthening effects, and thus overall the greenhouse gas forcing contribution since 1750 has been increased from 0.48 to 0.54 W/m^2, a 12.5% increase, without providing the opportunity of replicability of all assumptions made.

All in all, regarding all these multipliers, IPCC does not reveal the precise calculation path and modelling assumptions that went into computing the values. Readers of the IPCC report are being bounced back and forth between tables, chapters, sections and values, and then ultimately referred to some references that equally do not reveal the precise calculations and assumptions, so that in the end it is not possible to replicate the computations. That appears to violate scientific protocol.

B 7. The "potential" controversy

GW"Potential" is not the same as "Actual" atmospheric climate warming, which depends on several additional chemical and physical feedback cycles of planet Earth, which differ by the gases involved. Potential is mostly the result of modeling which is subject to numerous assumptions. There are still significant gaps between the "Potentials" that are being modeled and the "Actuals" that are observed, especially on regional levels (Laepple et al., 2023; https://www.carbonbrief.org/analysis-how-well-have-climate-models-projected-global-warming/; https://phys.org/news/2023-11-scientists-highlight-discrepancies-regional-climate.html)

B 8. The CO2 equivalency accounting controversies

There is considerable controversy around several different calculation methods that arrive at different CO_2 equivalency factors, such as GWP(x), GWP*, GTP(x) or ERF methods, and each of them need to be calculated over different time spans, such as 20, 50, 100 or 200 years. They are extensively explained in many other places, and therefore not replicated here. (IPCC AR 6, Chapter 7.6, pages 1011 - 1019).

B 9. The "feedback factor" controversy and popular "lifetime" misconception

Critical for understanding the impact of methane on atmospheric warming, is its lifetime. Between 90% and 93% of all atmospheric methane is destroyed by the radical hydroxyl (OH) (supplement A 2). The time span between emissions and destruction by such OH is described with atmospheric chemical lifetime. The longer the lifetime, the longer the methane is climate-warming effective. The IPCC AR6 value of 11.8 years which prompts the frequently mentioned "12 years" of methane lifetime in popular literature, is *not* the time span the CH4 remains on average in the atmosphere. Instead, that 11.8 years is what is called the so-called perturbation lifetime (PLT), which is lifetime multiplied with a factor of 1.3 in order to account for a feedback effect during the depletion of methane. Prominent estimations in recent years for average atmospheric chemical lifetimes of methane range from about six to ten years. In light of this broad range of estimates, the IPCC text converges on an atmospheric lifetime of 9.1 years (IPCC AR 6, Chapter 6.3.1, pp 835, 836; Stevenson et al 2020), without explaining why and how.

Popular discourse treats this "12 year" number like a life expectancy number from biology (as in dogs have an average lifespan of 12 years, so a group of 10 dogs will on average be gone after 12 years, but until then, most of the dogs will be alive for most of the time). However, in chemistry the average lifetime is defined as a so-called "e-fold time", which is the fraction factor by which the number *e* (=2.718) is exponentiated for a given time span, in order to reflect the exponential nature of the decay. The exponential nature would result from a Poisson distribution of likelihood of discrete independent chemical events occurring, which becomes lower and lower, the lesser of the original amount of decaying substance remains. This works out to about 37% of the initial substance still being left after one chemical lifetime has passed, and 14% after two lifetimes have passed and so on. The multiplication factor of 1.3 to arrive at PLT, shall reflect a presumed feedback effect where additional methane increases the concentrations which leads to longer lifetimes which leads to higher concentrations and so on (IPCC AR 6, Chapter 6.3.1, page 836). This assumption is based on early model calculations done in the 1990's (Prather, 2007) and reconfirmed on several similar premises thereafter (e.g. Stevenson et al., 2020) which, however, can be contested.

For instance, atmospheric chemistry regularly assumes in its modelling that the availability of OH for reactions with CH4 are "buffered", which means that they are largely independent of methane perturbations (Lelieveld et al., 2016). The buffering is the result of recycling OH mechanisms, which are generally underconsidered in atmospheric modelling (Turner 2017, Rigby 2017, Turner 2019). In this case, the feedback factor 1.3 would not be applicable.

B 10. The underlying Poisson distribution fallacy to the ~12-year perturbation lifetime

Unlike for instance in a radioactive decay process, the destruction process of methane in the atmosphere is not governed by a discrete Poisson distribution with independently occurring events, but is subject to multiple other factors such as temperature, pressure, weather patterns and presence of other atmospheric gases such as carbon monoxide (CO) or volatile organic compounds (VOC) that are competing with CH_4 for reaction availability with OH. Moreover, reactions might not be "using up" available OH, because depending on circumstances any given reaction might trigger the recreation of further OH for further reaction availability, in which case the central assumption of exponential e-folding would not apply to begin with, let alone a feedback factor. It is possible that the modelled lengthening of atmospheric lifetime with higher concentrations is entirely or mostly non-existent in reality, or even if they might be observed, that these two phenomena are unrelated with each other. Changes in methane lifetime might instead be dominated by changes in carbon monoxide (CO), nitrogen oxides (NO_x) or volatile organic compounds (VOC), three other atmospheric gases which are users and producers of OH in the atmosphere, and whose influences on OH are factor 3-5 stronger than methane (Lelieveld et al., 2016, Nguyen et al., 2020). In the words of the IPCC text: *"For example, the ERF attributed to methane emissions, which includes indirect effects through ozone formation and oxidation capacity with feedbacks on the methane lifetime, depend non-linearly on the concentrations of NOx, CO and NMVOCs. This means that the results from the model simulations depend to some extent on the chosen methodology."* (IPCC AR 6, Chapter 6.4.2, page 853). It should not be possible to claim a Poisson distribution of independent discrete events for one part of the methodology, and in a different part claim non-linear complexity as a shield against providing replicable data transparency.

B 11. The neglect of destruction delaying mechanisms in methane accounting

The amount and sources of CH4 emissions into the atmosphere are by themselves irrelevant to climate warming. Relevant are only the amounts of CH4 molecules residing in the atmosphere (the burden or the reservoir) which are acting as a greenhouse gas. Conceptually, the size of this CH4 reservoir is the result of a dynamic flow model of emission inflows, lifetime in the reservoir and destruction outflows. It helps to conceptualize these three factors in a stylized model as follows (figure S 7):

Figure S 7: Stylized model of methane in atmosphere

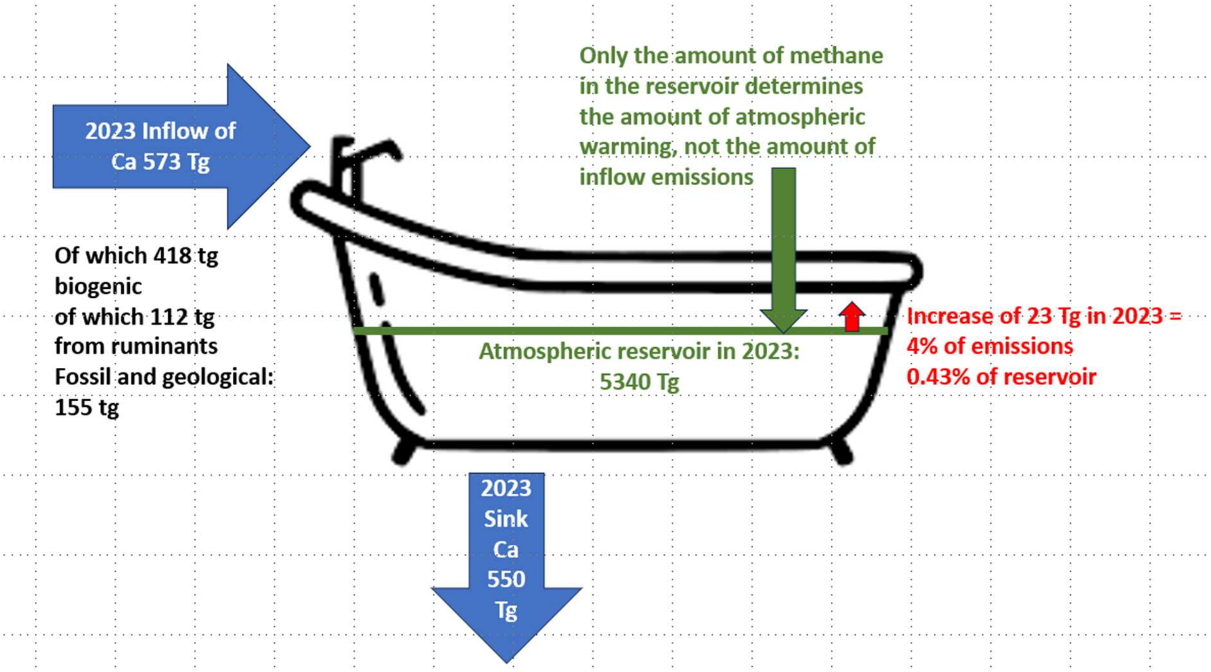

The following simplistic modelling shall illustrate the effect which delaying mechanisms have on the methane reservoir. (For comparison, the current annual amount of methane emissions is believed to be around 573 teragram, and the reservoir is 5340 teragram, see Supplement A 1):

Model A:

- Total emission: continuous and constant emissions of 100 Teragram (Tg) per year into the atmosphere
- Total destruction: destruction of 100 Tg per year
- Timing of destruction: instantaneous destruction at 12 noon each day of all available methane

In this model A, the methane would accumulate over a 24-hour period, and then be completely erased in one instance at noon. Thus, the average amount of methane reservoir during the year would be the daily emission divided by two, ie 100/365/2 = 0.137 Tg.

Model B:

- Total emission: continuous and constant emissions of 100 Teragram (Tg) per year into the atmosphere (same as model A)
- Total destruction: destruction of 100 Tg per year (same as model A)
- Due to a delaying mechanism in the atmosphere, instantaneous destruction only possible once per year on 31st of December of all available methane

In this model B, the methane would accumulate over a 365-day period, and then be completely erased in one instance on the last day of the year. Thus, the average amount of reservoir during the year would be 100/2 = 50 Tg.

The stark difference between model A and B shows that it is only driven by the timing of the methane destruction and thus its lifetime, not by the rates of respective emissions and destructions.

Model C:

- Continuous and constant emissions of 100 Teragram (Tg) per year into the atmosphere (same as model A)
- Due to a delaying mechanism, methane is destroyed only once per year on 31st of December (same as model B)
- In addition to the delaying mechanisms, due to an exogenous shock to the atmospheric system, only 90% of all prevalent methane can be destroyed.

In this model, there would be a carry-over of 10 Tg from the first year to the second year, and thus the average amount of reservoir during this second year would be 60 Tg. At the end of the year, of the then existing 110 Tg, again 90% would be destroyed, so 11 Tg will be carried over into the third year for an average total of 61 Tg etc.

The models illustrate, that the size of the reservoir is not primarily determined by the emissions, but mostly by the existence of delaying mechanisms which prevent the methane from being exposed to destruction processes, or the existence of exogenous shocks. For instance, if the delaying mechanism in model B is extended to two years, then the reservoir would increase to 100 Tg without any increase of emissions. Fluctuations of the size of the reservoir are therefore a lot more sensitive to any delaying mechanisms in place which extend the lifetime of methane, rather than the amount of emissions.

In real life, there are several mechanisms by which the destruction of methane is being delayed, with the main ones being:

- OH as the main reactant to CH_4 is created by a number of different reactions, most of which are related to the amount of sunlight and/or humidity present in the atmosphere. This favors the tropics and subtropics as the region where most OH is produced, and accordingly where most CH_4 is destroyed (see figure S 2). Around 70% of all methane is destroyed within the N 24° and S 24° latitudes. To the North, that is roughly the latitude of Miami, Dubai and Hong Kong. By contrast in the higher latitudes of 50° degrees (N and S) or more, only little OH is present, and therefore barely any methane becomes destroyed. Moreover, higher than the 30th latitudes, the prevailing wind regimes, especially in the winter, first carry the air and its CH_4 into the polar regions, where no methane destruction takes place. Eventually the air does pass back to the tropics and its CH4 will then be destroyed there, but until then, the CH_4 destruction has been delayed.
- 58% of the global methane emissions are emitted in the Northern Hemisphere north of 14° latitude, 9% south of 14°, and 33% in the tropical zones between N 14° and S 14°. Destruction of methane happens in equal amounts in the North and the South. However, it takes air from the North about a year until it is dissipated also to the South. Once arrived in the South, it might not immediately arrive in the tropics, but circulate around Antarctica (because it is transported there via the upper troposphere). Thus, the destruction of methane is delayed by the slow exchange of air between the Northern and Southern Hemispheres.
- In some areas above the equator, upward convection is so strong that air gets ejected into the stratosphere, where it will then circulate until eventually dissipating above the polar and sub-polar regions back into the troposphere. The residence time of air in the

stratosphere can be up to five years. While in the stratosphere, CH_4 methane gets destroyed much more slowly than in the troposphere, at an equivalent lifetime of 120 years only. Thus any CH_4 that escapes in the stratosphere is also delayed until it is eventually returned into the troposphere.

- CH_4 is not the only gas that reacts with OH. Only about 12% of available OH reacts with CH_4 (Lelieveld et al., 2016). The other main reactants are carbon monoxide (CO), and various volatile organic compounds (VOC). Thus the amounts of OH available to CH_4 can fluctuate with the amounts of CO and VOC that are prevalent in particular regions of the atmosphere. If these heightened concentrations of CO and VOC occur in the tropics, then it will be delaying the destruction of CH_4 overall because the chances of CH_4 to become destroyed when it cycles through tropical air, are accordingly diminished.

The average lifetime value of CH_4, regardless whether with a chemical or a biological meaning, hides considerable complexity. Depending on where the methane molecule is emitted, its chances of destruction timing are much different. A molecule emitted in the tropics or in the Southern hemisphere, is destroyed much faster than a molecule in the high Northern latitudes. Accordingly, the reduction of methane emissions might have a different impact on the decrease of the overall reservoir, depending on where they occur.

B 12. The unfortunate lack of transparency at IPCC

After all these possibly one-sided multiplications, amplifications and extensions for possible impacts of methane on global warming, the vast majority of which is based on modeling only for which a large array of assumptions can be made, and possible neglect of those factors that could be reducing methane's impact or alternative pathways of explanation, the IPCC AR 6 supposedly arrives at the value of 31% of all global warming forcing since 1750 being due to methane (in contrast to the a priori value of 13% mentioned above under Supplement B 3). As is explained in the previous section, the IPCC computation pathways are not transparent and not fully reflective of the many controversies on each of its components.

Moreover, these already complex IPCC values are frequently misquoted even in prominent publications. For instance, the already mentioned Saunois et al 2024 article which is supposed

to be a authoritative compilation of the global methane budget with a large authoring consortium, writes in its introduction: *"the emissions-based effective radiative forcing of CH4 concentrations has contributed ~31% (1.19 W m^{-2}) to the additional radiative forcing from anthropogenic emissions of greenhouse gases and their precursors (3.84 W m^{-2}) over the industrial era (1750-2019) (Forster et al 2021)"* (Forster et al = scientific citation for IPCC AR 6 Chapter 7).

Despite the prominence of its provenance, almost nothing in this Saunois et al sentence is correct. None of the IPCC reports mention the 3.84 W/m2 number. It can only be inferred from adding up a table in Chapter 6 (not chapter 7), which seems to speculate on a concept of "emissions-based effective radiative forcing" versus "abundance-based effective radiative forcing (IPCC AR 6, Chapter 6, Supplementary Material, page 3, already cited above under the section "Multiplication controversy"). However, it is not made clear in the IPCC texts how and where these emissions-based ERF's are being utilized (assumedly in the multiplier computations, but that is not clear). In chapter 7, to which Saunois et al refers to, the total anthropogenic emissions forcing is specified with the smaller value of 2.72 W/m2 (IPCC AR 6, Chapter 7, section 7.3.5.2, page 960), of which methane comprises 0.54 (IPCC AR 6, Chapter 7, section 7.3.2.2, pages 945-946), so a mere 20% instead of 31%.

Also, by definition, since the baseline for this 0.54 W/m2 value is chosen as 1750, it is assumed that any increase of concentrations since then must have been anthropogenic. That appears plausible at first sight. But then it is deduced that since the atmospheric concentrations have been rising since 1750, this entire rise must be related to increased emissions from human-caused sources of methane. That need not be the case. It is imaginable that the increase of concentrations is not related to "a" or "the" increase of emissions, but to a different change of dynamics of atmospheric chemistry, for instance a dramatic increase of lifetime, or a reduction of OH reaction capacity (see above Supplement B 11). It is not even clear that methane emissions today are higher than in the past to begin with (and compared to which past – the past of 1000, of 10,000 of 100,000 or one million years ago?). Such other dynamics would most likely also be human-caused, but then the mitigation and abatement path might have to look different. Instead of looking for human increases of methane emissions, one would have to look for human-caused changes in atmospheric dynamics. If it is different dynamics which are causing the increase in concentrations, then reducing emissions might have limited or even no

impact. That the cause of the increasing concentrations is due to higher levels of anthropogenic emissions since 1750, is a widespread assumption, but has never been uambiguously proven.

The frequent lop-sided and inaccurate quoting of IPCC material in the sciences and the peri-sciences, the IPCC texts itself being intransparent and its evaluations often non-replicable, and the choice of metrics that appear to maximize alarmism rather definitional clarity (by weight vs by molecules for instance), mirrors a debate where for instance all livestock related methane emissions are assigned to be anthropogenic, despite clear evidence that the paleolithic herd of ruminants emitted at least as much if not more methane, or that the much larger extent of rain forests and wetlands in paleolithic times must have also meant far higher methane emissions compared to today. It also mirrors a public policy discourse where beneficial impacts of ruminant livestock such as soil fertilization, carbon fixation in soils or biodiversity are either ignored or poorly accounted for (eg Manzano et al 2025).

All of which calls into question the reliability and the trust that can be placed into the scientific discourse (Ederer 2024).

References

Allen RJ, Zhao X, Randles CA, et al. Surface warming and wetting due to methane's long-wave radiative effects muted by short-wave absorption. Nat Geosci. 2023;16:314–320. doi: https://doi.org/10.1038/s41561-023-01144-z

Basu S, Lan X, Dlugokencky E, Michel S, Schwietzke S, Miller JB, Bruhwiler L, Oh Y, Tans PP, Apadula F, Gatti LV, Jordan A, Necki J, Sasakawa M, Morimoto S, Di Iorio T, Lee H, Arduini J, Manca G. Estimating emissions of methane consistent with atmospheric measurements of methane and δ13C of methane. Atmos Chem Phys. 2022;22:15351–15377. doi: https://doi.org/10.5194/acp-22-15351-2022.

Bey I, Jacob DJ, Yantosca RM, Logan JA, Field BD, Fiore AM, Li Q, Liu HY, Mickley LJ, Schultz MG. Global modeling of tropospheric chemistry with assimilated meteorology: Model description and evaluation. J Geophys Res. 2001;106(D19):23073–23095. doi:10.1029/2001JD000807

Bloom AA, Bowman KW, Lee M, Turner AJ, Schroeder R, Worden JR, Weidner R, McDonald KC, Jacob DJ. A global wetland methane emissions and uncertainty dataset for atmospheric chemical transport models (WetCHARTs version 1.0). Geosci Model Dev. 2017;10:2141–2156. doi: https://doi.org/10.5194/gmd-10-2141-2017.

Cheng H, Zhang H, Spötl C, Baker J, Sinha A, Li H, Bartolomé M, Moreno A, Kathayat G, Zhao J, Dong X, Li Y, Ning Y, Jia X, Zong B, Ait Brahim Y, Pérez-Mejías C, Cai Y, Novello VF, Cruz FW, Severinghaus JP, An Z, Edwards RL. Timing and structure of the Younger Dryas event and its underlying climate dynamics. Proc Natl Acad Sci U S A. 2020;117(38):23408–23417. doi: https://doi.org/10.1073/pnas.2007869117.

Conrad R. Methane production in soil environments—anaerobic biogeochemistry and microbial life between flooding and desiccation. Microorganisms. 2020;8(6):881. doi: https://doi.org/10.3390/microorganisms8060881.

Ederer P. Perspective on scientific truth versus scientific evidence; maintaining integrity in global food systems. Anim Prod Sci. 2024;64:AN23331. doi: http://doi.org/10.1071/AN23331.

EDGAR (Emissions Database for Global Atmospheric Research) Community GHG Database. A collaboration between the European Commission, Joint Research Centre (JRC), the International Energy Agency (IEA), and comprising IEA-EDGAR CO2, EDGAR CH4, EDGAR N2O, EDGAR F-GASES version 8.0. European Commission, JRC; 2023. Available from: https://edgar.jrc.ec.europa.eu/dataset_ghg80.

Ernst L, Steinfeld B, Barayeu U, et al. Methane formation driven by reactive oxygen species across all living organisms. Nature. 2022;603:482–487. doi: https://doi.org/10.1038/s41586-022-04511-9.

Fernandez-Cortes A, Cuezva S, Alvarez-Gallego M, et al. Subterranean atmospheres may act as daily methane sinks. Nat Commun. 2015;6:7003. doi: https://doi.org/10.1038/ncomms8003.

Ferretti DF, et al. Unexpected changes to the global methane budget over the past 2000 years. Science. 2005;309:1714–1717. doi: http://doi.org/10.1126/science.1115193.

Flood W. The methane misconceptions. Energy Environ. 2011;22(3):233–239. doi: http://www.jstor.org/stable/43735544.

Haller J. Carbon isotopes of methane during the Medieval Climate Anomaly [master's thesis]. Zurich (Switzerland): Swiss Federal Institute of Technology Zurich (ETHZ); 2013.

Herpen Mv, Li Q, Saiz-Lopez A, Liisberg JB, Röckmann T, Cuevas CA, et al. Photocatalytic chlorine atom production on mineral dust–sea spray aerosols over the North Atlantic. Proc Natl Acad Sci U S A. 2023;120(31):e2303974120. doi: https://doi.org/10.1073/pnas.2303974120.

Intergovernmental Panel on Climate Change (IPCC). Climate change 2013: The physical science basis. Chapter 8: Anthropogenic and natural radiative forcing [Internet]. IPCC AR5 Working Group I Report. 2013. Available from: https://www.ipcc.ch/site/assets/uploads/2018/02/WG1AR5_Chapter08_FINAL.pdf

Intergovernmental Panel on Climate Change (IPCC). Climate change 2013: The physical science basis. Chapter 8: Anthropogenic and natural radiative forcing. Supplementary material [Internet]. IPCC AR5 Working Group I Report. 2013. Available from: https://www.ipcc.ch/site/assets/uploads/2018/07/WGI_AR5.Chap_.8_SM.pdf

Intergovernmental Panel on Climate Change (IPCC). Climate change 2021: The physical science basis. Chapter 6: Short-lived climate forcers [Internet]. IPCC AR6 Working Group I Report. 2021. Available from: https://www.ipcc.ch/report/ar6/wg1/downloads/report/IPCC_AR6_WGI_Chapter06.pdf

Intergovernmental Panel on Climate Change (IPCC). Climate change 2021: The physical science basis. Chapter 6: Short-lived climate forcers. Supplementary material. IPCC AR6 Working Group I Report. 2021. Available from: https://www.ipcc.ch/report/ar6/wg1/downloads/report/IPCC_AR6_WGI_Chapter06_SM.pdf

Intergovernmental Panel on Climate Change (IPCC). Climate change 2021: The physical science basis. Chapter 7: The ocean and cryosphere in a changing climate. IPCC AR6 Working Group I Report. 2021. Available from: https://www.ipcc.ch/report/ar6/wg1/chapter/chapter-7/

Intergovernmental Panel on Climate Change (IPCC). Climate change 2021: The physical science basis. Chapter 7: The ocean and cryosphere in a changing climate. Supplementary material. IPCC AR6 Working Group I Report. 2021. Available from: https://www.ipcc.ch/report/ar6/wg1/downloads/report/IPCC_AR6_WGI_Chapter07_SM.pdf

Intergovernmental Panel on Climate Change (IPCC). Climate change 2021: Mitigation of climate change. Annex III: Greenhouse gas emissions reduction potentials and costs. IPCC AR6 Working Group III Report. 2021. Available from: https://www.ipcc.ch/report/ar6/wg3/downloads/report/IPCC_AR6_WGIII_Annex-III.pdf

Khalil MAK, Butenhoff CL, Rasmussen RA. Atmospheric methane: Trends and cycles of sources and sinks. Environ Sci Technol. 2007;41(7):2131–2137. doi: https://doi.org/10.1021/es061791t .

Laepple T, Ziegler E, Weitzel N, et al. Regional but not global temperature variability underestimated by climate models at supradecadal timescales. Nat Geosci. 2023;16:958–966. doi: https://doi.org/10.1038/s41561-023-01299-9.

Lan X, Basu S, Schwietzke S, Bruhwiler LMP, Dlugokencky EJ, Michel SE, et al. Improved constraints on global methane emissions and sinks using δ13C-CH4. Global Biogeochem Cycles. 2021;35:e2021GB007000. doi: https://doi.org/10.1029/2021GB007000.

Lelieveld, J., Gromov, S., Pozzer, A., and Taraborrelli, D.: Global tropospheric hydroxyl distribution, budget and reactivity, Atmos. Chem. Phys., 16, 12477–12493, https://doi.org/10.5194/acp-16-12477-2016 , 2016.

Luo et al. The Global Methane Budget I Poster. AGU Fall Meeting 2023, San Francisco, CA, 11-15 December 2023. Poster No. 2108, Session: Biogeosciences. Abstract available from: https://ui.adsabs.harvard.edu/abs/2023AGUFM.B21K2108L/abstract.

Loulergue L, Schilt A, Spahni R, Masson-Delmotte V, Blünier T, Lemieux B, Barnola JM, Raynaud D, Stocker TF, Chappellaz J. Orbital and millennial-scale features of atmospheric CH4 over the past 800,000 years. Nature. 2008;453:383–386. doi: https://doi.org/10.1038/nature06950.

Manzano P, De Aragão Pereira M, and Windisch W. Vast extension but positive outcomes, reduced but negative: complexity and nuances in evaluating land use by livestock and crops. 2025. Animal Frontiers Vol 15, Issue 1

Mao Y, Tong L, Li H, He R, Ye K, Yu W, He Q. Aerobic methane production by phytoplankton as an important methane source of aquatic ecosystems: Reconsidering the global methane budget. Sci Total Environ. 2024;907:167864. doi: https://doi.org/10.1016/j.scitotenv.2023.167864.

Naughton F, Sánchez-Goñi MF, Landais A, Rodrigues T, Vazquez Riveiros N, Toucanne S. Chapter 6 - The Bølling–Allerød Interstadial. In: Palacios D, Hughes PD, García-Ruiz JM, Andrés N, editors. European glacial landscapes. Elsevier; 2023. p. 45–50. doi: https://doi.org/10.1016/B978-0-323-91899-2.00015-2.

Nelson RC, Plyler EK, Benedict WS. Absorption spectra of methane in the near infrared. J Res Natl Bur Stand. 1948;41:615.

Nguyen NH, Turner AJ, Yin Y, Prather MJ, Frankenberg C. Effects of chemical feedbacks on decadal methane emissions estimates. Geophys Res Lett. 2020;47:e2019GL085706. doi: https://doi.org/10.1029/2019GL085706.

Nisbet EG, Manning MR, Dlugokencky EJ, Michel SE, Lan X, Rockmann T, et al. Atmospheric methane: Comparison between methane's record in 2006–2022 and during glacial terminations. Global Biogeochem Cycles. 2023;37:e2023GB007875. doi: https://doi.org/10.1029/2023GB007875.

Oh Y, Zhuang Q, Welp LR, et al. Improved global wetland carbon isotopic signatures support post-2006 microbial methane emission increase. Commun Earth Environ. 2022;3:159. doi: https://doi.org/10.1038/s43247-022-00488-5.

Oh Y, Bruhwiler L, Lan X, Basu S, Schuldt K, Thoning K, Michel SE, Clark R, Miller JB, Andrews A, Sherwood O, Etiope G, Crippa M, Liu L, Zhuang Q, Randerson J, van der Werf G, Aalto T, Amendola S, et al. CarbonTracker CH4 2023. NOAA. Accessed November 2024.

Prather MJ. Lifetimes and time scales in atmospheric chemistry. Philos Trans A Math Phys Eng Sci. 2007;365:1705–1726. doi: https://doi.org/10.1098/rsta.2007.2040.

Prather M. The nonlinear nature of atmospheric chemistry. EGU General Assembly 2021, online, 19–30 Apr 2021. EGU21-8934. doi: https://doi.org/10.5194/egusphere-egu21-8934.

Quiquet A, Archibald AT, Friend AD, Chappellaz J, Levine JG, Stone EJ, Telford PJ, Pyle JA. The relative importance of methane sources and sinks over the Last Interglacial period and into the last glaciation. Quat Sci Rev. 2015;112:1–16. doi: https://doi.org/10.1016/j.quascirev.2015.01.004.

Rigby M, Montzka SA, Prinn RG, White JWC, Young D, O'Doherty S, Lunt MF, Ganesan AL, Manning AJ, Simmonds PG, Salameh PK, Harth CM, Muhle J, Weiss RF, Fraser PJ, Steele LP, Krummel PB, McCulloch A, Park S. Role of atmospheric oxidation in recent methane growth. P Natl Acad Sci U S A. 2017;114:5373–5377. doi: https://doi.org/10.1073/pnas.1616426114.

Rubino M, Etheridge DM, Thornton DP, Howden R, Allison CE, Francey RJ, Langenfelds RL, Steele LP, Trudinger CM, Spencer DA, Curran MAJ, van Ommen TD, Smith AM. Revised records of atmospheric trace gases CO2, CH4, N2O, and δ13C-CO2 over the last 2000 years from Law Dome, Antarctica. Earth Syst Sci Data. 2019;11:473–492. doi: https://doi.org/10.5194/essd-11-473-2019.

Sander R, Baumgaertner A, Cabrera-Perez D, Frank F, Gromov S, Grooß JU, Harder H, Huijnen V, Jöckel P, Karydis VA, Niemeyer KE, Pozzer A, Riede H, Schultz MG, Taraborrelli D, Tauer S. The community atmospheric chemistry box model CAABA/MECCA-4.0. Geosci Model Dev. 2019;12:1365–1385. doi: https://doi.org/10.5194/gmd-12-1365-2019.

Saunois M, Martinez A, Poulter B, Zhang Z, Raymond P, Regnier P, Canadell JG, Jackson RB, Patra PK, Bousquet P, Ciais P, Dlugokencky EJ, Lan X, Allen GH, Bastviken D, Beerling DJ, Belikov DA, Blake DR, Castaldi S, Crippa M, Deemer BR, Dennison F, Etiope G, Gedney N, Höglund-Isaksson L, Holgerson MA, Hopcroft PO, Hugelius G, Ito A, Jain AK, Janardanan R, Johnson MS, Kleinen T, Krummel P, Lauerwald R, Li T, Liu X, McDonald KC, Melton JR, Mühle J, Müller J, Murguia-Flores F, Niwa Y, Noce S, Pan S, Parker RJ, Peng C, Ramonet M, Riley WJ, Rocher-Ros G, Rosentreter JA, Sasakawa M, Segers A, Smith SJ, Stanley EH, Thanwerdas J, Tian H, Tsuruta A, Tubiello FN, Weber TS, van der Werf G, Worthy DE, Xi Y, Yoshida Y, Zhang W, Zheng B, Zhu Q, Zhu Q, Zhuang Q. Global methane budget 2000–2020. Earth Syst Sci Data Discuss. 2024. In review. doi: https://doi.org/10.5194/essd-2024-115.

Selvam AM. Nonlinear dynamics and chaos: Applications in atmospheric. 2010. Available from: https://arxiv.org/pdf/1006.4554.

Smith FA, Hammond JI, Balk MA, Elliott SM, Lyons SK, Pardi MI, Tomé CP, Wagner PJ, Westover ML. Exploring the influence of ancient and historic megaherbivore extirpations on the global methane budget. P Natl Acad Sci U S A. 2016;113:874–879. doi: https://doi.org/10.1073/pnas.1502547112.

Snyder, C.W. 2016. Evolution of global temperature over the past two million years. Nature, Vol. 538, pp. 226-228.

Soni M, Sander R, Sahu LK, Taraborrelli D, Liu P, Patel A, Girach IA, Pozzer A, Gunthe SS, Ojha N. Comprehensive multiphase chlorine chemistry in the box model CAABA/MECCA: implications for atmospheric oxidative capacity. Atmos Chem Phys. 2023;23:15165–15180. doi: https://doi.org/10.5194/acp-23-15165-2023.

Stevenson DS, Zhao A, Naik V, O'Connor FM, Tilmes S, Zeng G, Murray LT, Collins WJ, Griffiths PT, Shim S, Horowitz LW, Sentman LT, Emmons L. Trends in global tropospheric hydroxyl radical and methane lifetime since 1850 from AerChemMIP. Atmos Chem Phys. 2020; 20:12905–12920. doi: https://doi.org/10.5194/acp-20-12905-2020.

Tedeschi LO, Beauchemin KA. Galyean appreciation club review: A holistic perspective of the societal relevance of beef production and its impacts on climate change. J Anim Sci. 2023;101:skad024. doi: https://doi.org/10.1093/jas/skad024.

Turner AJ, Frankenberg C, Wennberg PO, Jacob DJ. Ambiguity in the causes for decadal trends in atmospheric methane and hydroxyl. P Natl Acad Sci U S A. 2017;114:5367–5372. doi: https://doi.org/10.1073/pnas.1616020114.

Turner AJ, Jacob DJ, Benmergui J, Brandman J, White L, Randles CA. Assessing the capability of different satellite observing configurations to resolve the distribution of methane emissions at kilometer scales. Atmos Chem Phys. 2018;18:8265–8278. doi: https://doi.org/10.5194/acp-18-8265-2018.

Turner AJ, Frankenberg C, Kort EA. 2019. Assessing methane emissions from space. Proc Natl Acad Sci U S A. 2019;116:2805–2813. doi: https://doi.org/10.1073/pnas.1814297116.

Wilkinson DM, Nisbet EG, Ruxton GD. Could methane produced by sauropod dinosaurs have helped drive Mesozoic climate warmth? Curr Biol. 2012;22(9):R292–R293. doi: https://doi.org/10.1016/j.cub.2012.03.042.

Wu W, Liu X, Xiong X, Yang Q, Zhou L, Lei L, Zhou DK, Larar AM. Spectral fingerprinting of methane from hyper-spectral sounder measurements using machine learning and radiative kernel-based inversion. Remote Sens. 2024;16:578. doi: https://doi.org/10.3390/rs16030578.

Yang X, Guo L, Zheng Z, Riemer N, Tessum CW. Atmospheric chemistry surrogate modeling with sparse identification of nonlinear dynamics. J Geophys Res: Machine Learn Comput. 2024;1:e2024JH000132. doi: https://doi.org/10.1029/2024JH000132.

Yin Y, Chevallier F, Ciais P, Bousquet P, Saunois M, Zheng B, Worden J, Bloom AA, Parker RJ, Jacob DJ, Dlugokencky EJ, Frankenberg C. Accelerating methane growth rate from 2010 to 2017: leading contributions from the tropics and East Asia. Atmos Chem Phys. 2021;21:12631–12647. doi: https://doi.org/10.5194/acp-21-12631-2021.

Zhu, S., Feng, L., Liu, Y. et al. Decadal Methane Emission Trend Inferred from Proxy GOSAT XCH4 Retrievals: Impacts of Transport Model Spatial Resolution. Adv. Atmos. Sci. 39, 1343–1359 (2022). https://doi.org/10.1007/s00376-022-1434-6.

Zhuang J, Jacob DJ, Flo-Gaya J, Yantosca RM, Lundgren EW, Sulprizio MP, Eastham SD. Enabling immediate access to Earth science models through cloud computing: application to the GEOS-Chem model. Bull Am Meteorol Soc. 2019;100:1943–1960. doi: https://doi.org/10.1175/BAMS-D-18-0243.1.
